# Function of multiple sclerosis-protective HLA class I alleles revealed by genome-wide protein-quantitative trait loci mapping of interferon signalling

**DOI:** 10.1371/journal.pgen.1009199

**Published:** 2020-10-26

**Authors:** Christian Lundtoft, Pascal Pucholt, Juliana Imgenberg-Kreuz, Jonas Carlsson-Almlöf, Maija-Leena Eloranta, Ann-Christine Syvänen, Gunnel Nordmark, Johanna K. Sandling, Ingrid Kockum, Tomas Olsson, Lars Rönnblom, Niklas Hagberg

**Affiliations:** 1 Rheumatology and Science for Life Laboratories, Department of Medical Sciences, Uppsala University, Uppsala, Sweden; 2 Molecular Medicine and Science for Life Laboratory, Department of Medical Sciences, Uppsala University, Uppsala, Sweden; 3 Centre for Molecular Medicine, Department of Clinical Neuroscience, Karolinska Institutet, Stockholm, Sweden; The Jackson Laboratory, UNITED STATES

## Abstract

Interferons (IFNs) are cytokines that are central to the host defence against viruses and other microorganisms. If not properly regulated, IFNs may contribute to the pathogenesis of inflammatory autoimmune, or infectious diseases. To identify genetic polymorphisms regulating the IFN system we performed an unbiased genome-wide protein-quantitative trait loci (pQTL) mapping of cell-type specific type I and type II IFN receptor levels and their responses in immune cells from 303 healthy individuals. Seven genome-wide significant (p < 5.0E-8) pQTLs were identified. Two independent SNPs that tagged the multiple sclerosis (MS)-protective HLA class I alleles A*02/A*68 and B*44, respectively, were associated with increased levels of IFNAR2 in B and T cells, with the most prominent effect in IgD^–^CD27^+^ memory B cells. The increased IFNAR2 levels in B cells were replicated in cells from an independent set of healthy individuals and in MS patients. Despite increased IFNAR2 levels, B and T cells carrying the MS-protective alleles displayed a reduced response to type I IFN stimulation. Expression and methylation-QTL analysis demonstrated increased mRNA expression of the pseudogene *HLA-J* in B cells carrying the MS-protective class I alleles, possibly driven via methylation-dependent transcriptional regulation. Together these data suggest that the MS-protective effects of HLA class I alleles are unrelated to their antigen-presenting function, and propose a previously unappreciated function of type I IFN signalling in B and T cells in MS immune-pathogenesis.

## Author summary

Genetic association studies have been very successful in identifying disease-associated single nucleotide polymorphisms (SNPs), but it has been challenging to define the molecular mechanisms underlying these associations. As interferons (IFNs) have a central role in the immune system, we hypothesized that some of the SNPs associated to immune-mediated diseases would affect the IFN system. By combining genetic data with characterization of interferon receptor levels and their responses on the protein level in immune cells from 303 genotyped healthy individuals, we show that two SNPs tagging the HLA class I alleles A*02/A*68 and B*44 are associated with a decreased response to type I IFN stimulation in B cells and T cells. Notably, both HLA-A*02 and HLA-B*44 confer protection from developing multiple sclerosis (MS), which is a chronic inflammatory neurologic disease. In addition to suggesting a pathogenic role of enhanced type I interferon signalling in B cells and T cells in MS, our data emphasize the fact that genetic associations in the HLA locus can affect functions not directly associated to antigen presentation, which conceptually may be important for other diseases genetically associated to the HLA locus.

## Introduction

Interferons (IFNs) are cytokines central for the immune response against viruses, bacteria and certain malignancies [1, 2]. The production and response to IFNs are strictly controlled, since an inappropriate activation of the IFN system can lead to the break of immune tolerance and the development of autoimmune diseases [3]. Based on receptor usage, the IFNs can be grouped into three different subclasses. Type I IFNs (including IFN-α/β), signal through the ubiquitously expressed IFNα/β-receptor (IFNAR). Type II IFN (IFN-γ) signals through the IFN-γ receptor (IFNGR) mainly expressed by monocytes, B cells and T cells, whereas type III IFNs (IFN-λ) signal through the IFN-λ receptor primarily expressed by epithelial cells [4]. IFN receptor signaling is transmitted via Janus kinas-mediated phosphorylation of transcription factors belonging to the signal transducer and activator of transcription (STAT) family, which drive the transcription of a large number of genes, including chemokines and HLA molecules [5].

Genome-wide association studies (GWAS) have identified thousands of inflammatory disease-associated single nucleotide polymorphisms (SNPs) [6] and many of these are located in genes connected to the IFN system [7]. The majority of disease-associated SNPs are located in non-coding regions of the genome and the underlying molecular mechanisms are mostly unknown. Data from genome-wide expression quantitative trait loci (eQTL) studies demonstrate that the regulatory effects of genetic variants are often context-dependent and cell-type specific [8–12]. Because protein levels to a large extent are buffered against transcriptional variation through post-transcriptional regulatory mechanisms, the degree to which an eQTL translates to a corresponding protein QTL (pQTL) or conversely is highly variable [13]. Hence, it is important to complement eQTL studies with systematic studies of how genetic variation influences protein levels. Several large-scale genome-wide pQTL studies have analyzed the genetic regulation of frequencies of immune cell subsets, cell-surface protein expression and the plasma proteome during homeostatic conditions [14–20], but genome-wide context-dependent and cell-type specific pQTL-studies are more limited [21, 22].

Despite the large number of disease associations in genes linked to the IFN system, and the fact that genetic variants contributing to a balanced IFN response probably have been positively selected for during evolution, there has been no comprehensive study to investigate the genetic regulation of the type I and type II IFN receptor responses on the protein level. In this study we performed a genome-wide cell type-specific pQTL mapping of 45 IFN related traits in six subsets of immune cells from 303 healthy individuals.

## Results

### Protein QTL mapping of IFN-related traits identifies seven novel pQTLs

**Fig 1:**
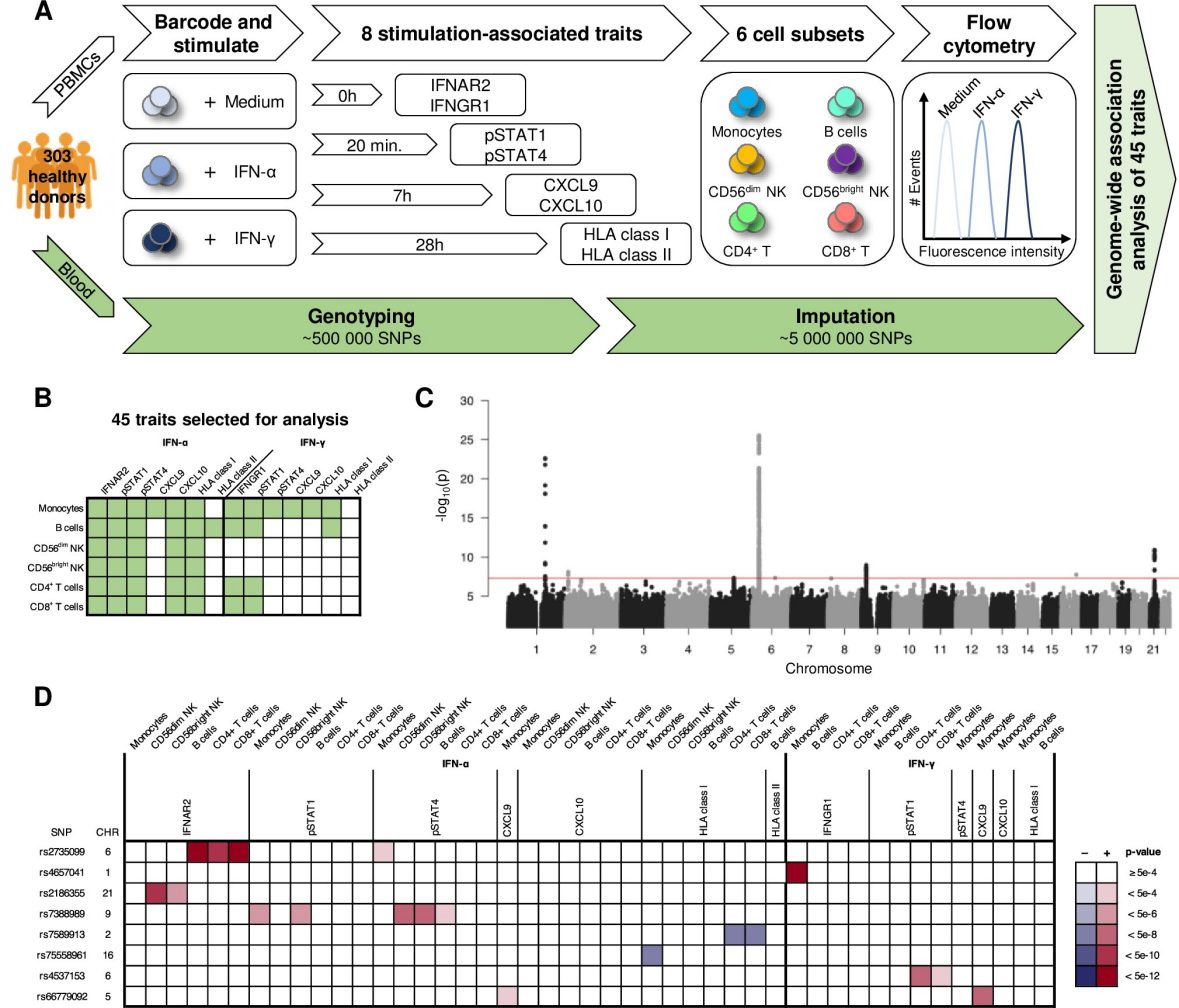
pQTL analysis of IFN-α and IFN-γ receptor levels and responses. (A) Schematic overview of the study design. For detailed experimental procedures see the Methods section. (B) Depiction of the 45 traits selected for pQTL analysis marked in green. (C) A combined Manhattan plot of the 45 pQTL analyses with the lowest p-value for each SNP depicted in the plot. The red line denotes the genome-wide significance level of p < 5.0E-8. (D) A heat-map with p-values of the eight genome-wide significant SNPs for all 45 traits studied. Blue indicates a decrease in minor allele carriers, while red indicates an increase in minor allele carriers. (C and D) p-values from the full model as described in the Methods section. (*n* = 303).

To study the effects of genetic variation on the type I and type II IFN systems, we analysed cell surface levels of the ligand binding high-affinity subunit of the IFN-α/β receptor (IFNAR2) and the ligand-binding subunit of the IFN-γ receptor (IFNGR1) as well as down-stream effects of receptor ligation, such as phosphorylation of transcription factors (pSTAT1 and pSTAT4), chemokine production (CXCL9 and CXCL10), and HLA class I and HLA class II receptor levels in six immune cell subsets in PBMCs from 303 healthy individuals using flow cytometry (Fig 1A). Based on surface levels of IFN receptors and the IFN-responsiveness in different cell types, 45 traits were selected for further studies (Fig 1B and S1 Fig). Genome-wide association analysis of these 45 traits identified eight independent genome-wide significant pQTLs (p < 5.0E-8; Fig 1C and Table 1; S2 Data all variants with a p-value < 1.0E-4). Three of the pQTLs regulated IFN receptor levels, whereas five pQTLs controlled IFN receptor responses. There was no overlap between IFN-α and IFN-γ induced pQTLs and the majority of pQTLs were cell-type specific (Fig 1D). Furthermore, of the eight pQTLs, one was a *cis*-pQTL and seven were *trans*-pQTLs. All pQTLs were located in non-coding regions of the genome, but one SNP was in complete LD with a missense SNP in *FCGR2A* (rs1801274, His167Arg) previously associated with systemic lupus erythematosus [23], ulcerative colitis [24] and Kawasaki disease [25]. Follow-up studies revealed that this pQTL was a false positive due to differential Fc-receptor binding of the anti-IFNGR1 mAb (S2 Fig) [26]. This finding stresses the fact that genetic associations between SNPs in the Fc receptor locus and traits determined using antibodies [14–16] should be interpreted cautiously and preferably validated using antibodies without Fc-binding properties.

**Table 1:** Summary of genome-wide significant pQTLs.

SNP	CHR	MAF	p	Trait	Stimulation	Cell type	Gene	Location	Imputation score
rs2735099	6	0.42	3.0E-26	IFNAR2	None	B cells	*HLA-A*	1.2 kb 3' of *HLAA*	0.98
rs4657041*	1	0.46	2.5E-23	IFNGR1	None	Monocytes	*FCGR2A*	Intronic	0.99
rs2186355	21	0.14	1.3E-11	IFNAR2	None	CD56^dim^ NK	*IFNAR2*	21 kb 5' of *IFNAR2*	Genotyped
rs7388989	9	0.29	1.1E-09	pSTAT4	IFNα	CD56^dim^ NK	*FOCAD*	Intronic	1.00
rs7589913	2	0.17	8.2E-09	HLA-ABC	IFNα	CD8^+^ T cells	*LOC100506457*	333 kb 5' of *TRIB2*	0.98
rs75558961	16	0.05	1.8E-08	HLA-ABC	IFNα	Monocytes	*CDH11*	Intronic	0.95
rs4537153	6	0.08	4.6E-08	pSTAT1	IFNγ	CD4^+^ T cells	.	143 kb 3' of *MCHR2*	0.98
rs66779092	5	0.18	4.7E-08	CXCL9	IFNγ	Monocytes	.	338 kb 3' of *SLCO4C1*	0.98

*Identified as a false positive in subsequent studies

In summary, we identify seven independent pQTLs regulating IFN receptor expression levels or IFN receptor responses.

### Multiple *cis*-pQTLs regulate IFNAR2 surface levels in a cell-type specific manner

**Fig 2:**
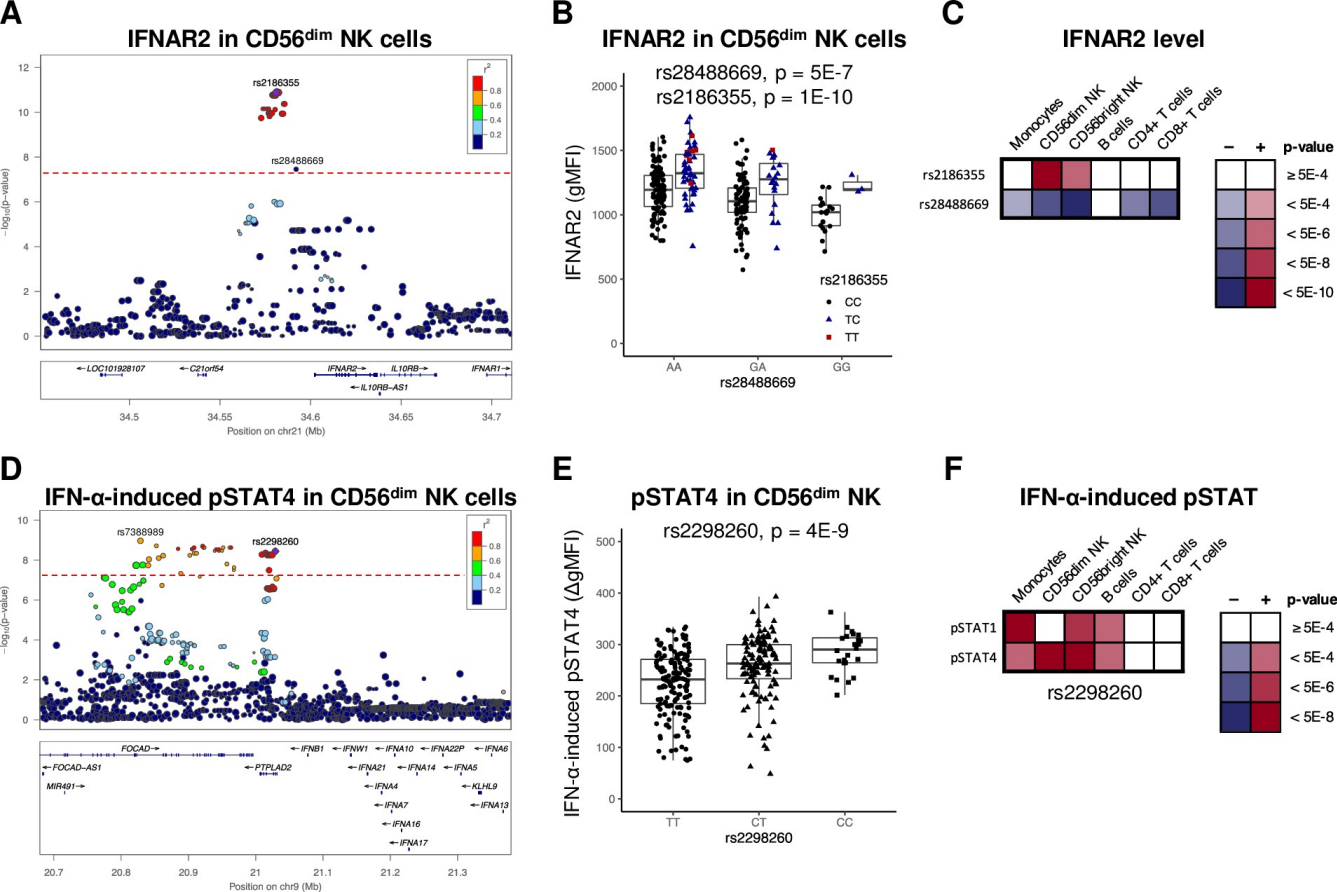
Cell-type and trait specific effects of pQTLs. (A-C) Data on IFNAR2 cell surface levels. (A) Regional association plot for IFNAR2 surface levels in CD56^dim^ NK cell and (B) boxplots stratified by rs28488669 and rs2186355. (C) Heat-map of the relation between p-values for the two *cis*-IFNAR2 pQTLs in different cell-types as specified. (D-E) Data on IFN-α induced phosphorylation of STAT4 (pSTAT4) and STAT1 (pSTAT1). (D) Regional association plot of the IFN-α induced pSTAT4 in CD56^dim^ NK cells and (E) boxplots stratified by rs2298260. (F) Heat-map of the relation between pQTL p-values for IFN-α induced pSTAT1 and pSTAT4 in indicated cell-types. (A and D) The genome-wide significance level (p < 5.0E-8) is denoted by a red line. (A-F) p-values from the full model using single (A, D-F), or two additive SNPs (B-C), (*n* = 303). Boxplots show median, IQR and range. gMFI = geometric mean fluorescence intensity.

The only genome-wide significant *cis*-pQTL identified was an association between rs2186355 located upstream of *IFNAR2* and IFNAR2 surface protein levels in CD56^dim^ NK cells (p = 1.3E-11). A closer analysis of this locus revealed a second independent pQTL at rs28488669 regulating IFNAR2 levels in CD56^dim^ NK cells in an additive manner (Fig 2A and 2B, p-value for test of interaction = 0.41). While the effect of rs2186355 was specific for CD56^dim^ and CD56^bright^ NK cells, rs28488669 also regulated IFNAR2 surface levels in CD8^+^ and CD4^+^ T cells (p < 5.0E-6; Fig 2C and S3 Fig). Both genetic variants have previously been reported as *cis*-eQTLs for *IFNAR2* mRNA expression in whole blood [27], but to the best of our knowledge, this is the first time that these SNPs have been associated with IFNAR2 at the protein level and in a cell-type specific manner.

### A pQTL in the *FOCAD* locus associates with an increased type I IFN response in NK cells

The strongest pQTL for an IFN-induced trait was the association between the intronic SNP rs7388989 in *FOCAD* and IFN-α-induced pSTAT4 in CD56^dim^ NK cells (p = 1.1E-9). This SNP is in LD (r^2^ = 0.79) with the missense SNP rs2298260 (Thr36Ala) in *HACD4* (previously named *PTPLAD2*), which in addition to IFN-α-induced pSTAT4 in CD56^dim^ NK cells (p = 3.6E-9) was associated with IFN-α-induced pSTAT4 in CD56^bright^ NK cells as well as IFN-α-induced pSTAT1 in monocytes and CD56^bright^ NK cells (p < 5.0E-6; Fig 2D–2F, and S4A and S4B Fig). Conditional analysis showed that both SNPs represented the same association signal (S4C Fig). The increased IFN-α-response in monocytes from minor allele carriers of rs2298260 translated into an increased production of CXCL9 (p = 1.1E-3; S3D Fig), whereas no effect was observed for CXCL10 (p = 0.94; S3E Fig). A SNP in complete LD with rs2298260 (rs2275888, r^2^ = 1.0) was previously identified as a *cis*-eQTL for *IFNB1* in LPS-stimulated monocytes, which drove a *trans*-eQTL network consisting of multiple IFN-response genes [8]. Importantly, in contrast to the increased IFN-α-induced pSTAT4/pSTAT1 observed in minor allele carriers of rs2298260 in our study, the LPS-induced expression of *IFNB1* was reduced in minor allele carriers in that study. Together these data highlight the importance of context-specific effects of regulatory SNPs.

### B cells and T cells carrying the MS-protective HLA-A*02, A*68 and B*44 alleles have increased IFNAR2 surface levels in B cells and T cells

**Fig 3:**
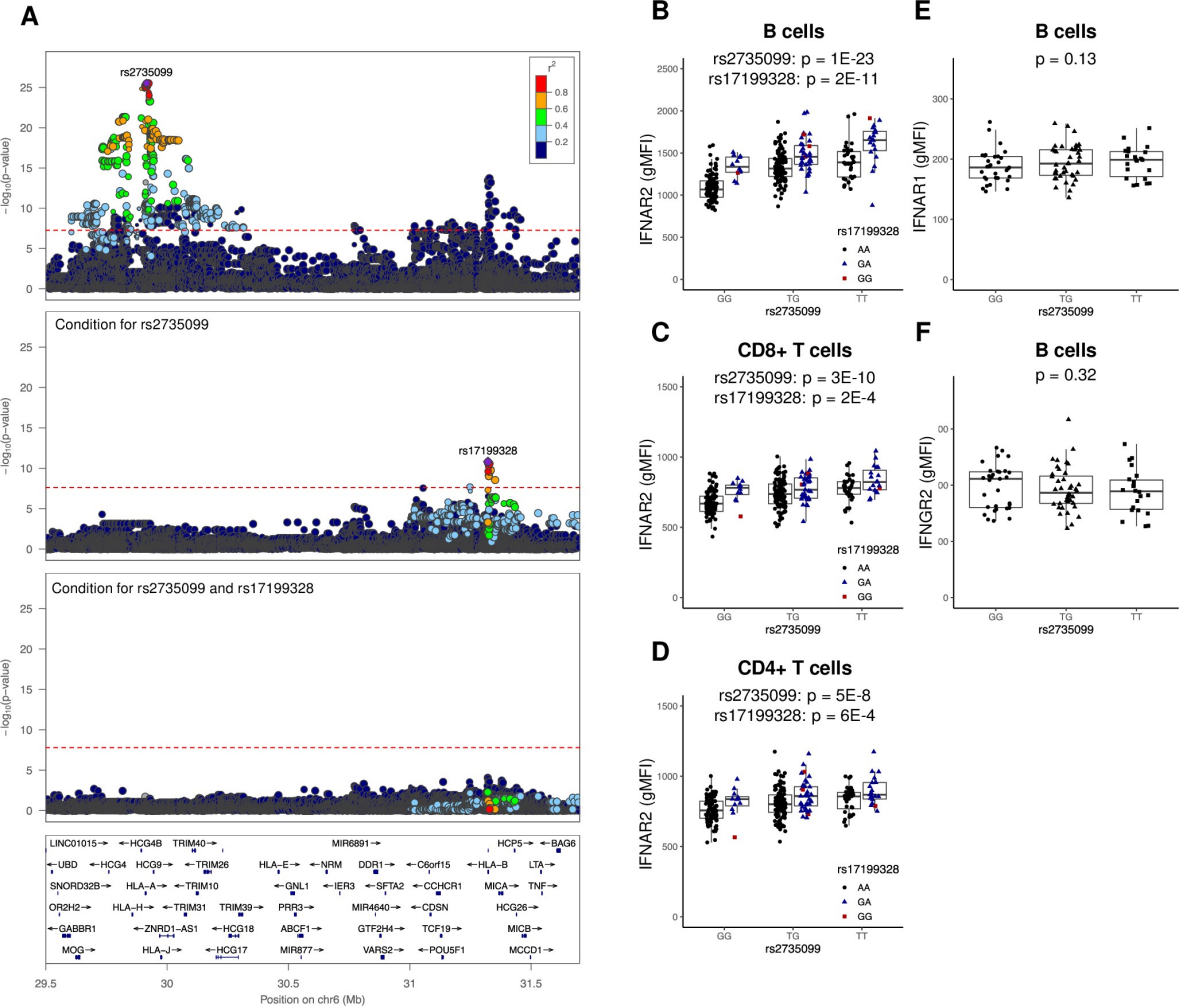
Two independent SNPs additively associate with IFNAR2 protein levels in B cells and T cells. (A) Regional association plots of the *trans*-IFNAR2 pQTL in B cells before (top) and after conditioning for rs2735099 (middle) and rs17199328 (bottom). (B-D) Boxplots of IFNAR2 levels in B cells (B), CD8^+^ T cells (C) and CD4^+^ T (D) cells stratified by rs2735099 and rs17199328 (*n* = 301). (E-F) IFNAR1 (E) and IFNGR2 (F) surface levels in B cells (*n* = 95). p-values from the full model using single (A, E and F), or two additive SNPs (B-D). Boxplots show median, IQR and range. gMFI = geometric mean fluorescence intensity.

The top pQTL was a *trans*-association between rs2735099 located 1.2 kb downstream of *HLA-A* on chromosome 6 and IFNAR2 surface levels in B cells (p = 3.0E-26; Fig 3A, top panel). Conditioning on rs2735099 revealed a second independent signal from the intronic SNP rs17199328 located in *HLA-B* (p = 1.6E-11; Fig 3A, middle panel). The effect of rs2735099 and rs17199328 was additive with minor allele carriers displaying increased B cell IFNAR2 surface levels (Fig 3B). Similarly, increased IFNAR2 surface levels were seen in CD8^+^ and CD4^+^ T cells from minor allele carriers of the two SNPs (Fig 3C and 3D), but not in NK cells or monocytes (p_CD56dim_ = 0.05, p_CD56bright_ = 0.15, p_monocyte_ = 0.13; S5A Fig). As the genes encoding IFNAR1 and IFNGR2 are located adjacent to *IFNAR2*, we investigated the possibility of co-regulatory effects, but neither IFNAR1 nor IFNGR2 surface levels in B cells were associated with rs2735099 or rs17199328 (p_IFNAR1_ = 0.13, p_IFNGR2_ = 0.32, *n* = 95; Fig 3E and 3F).

**Fig 4:**
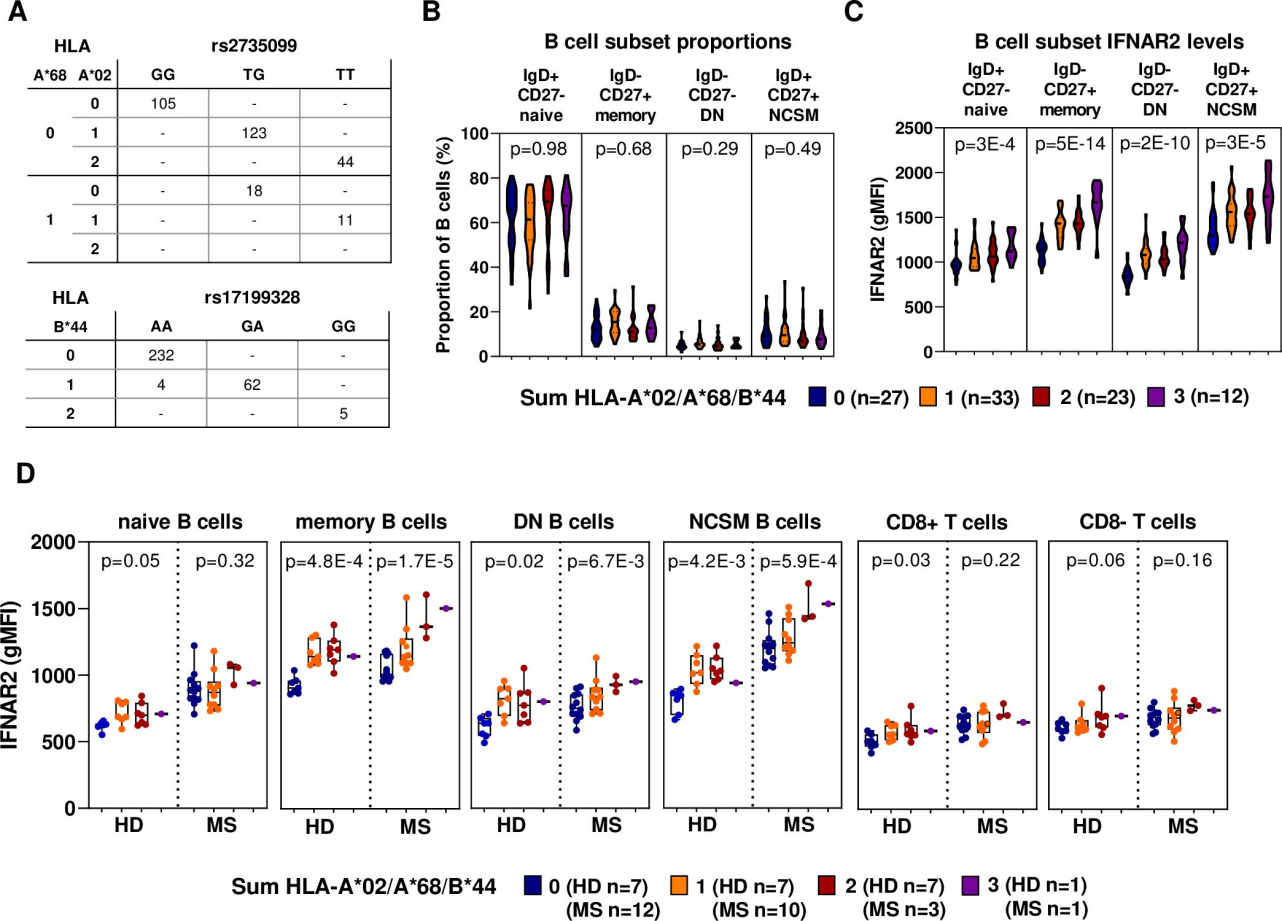
Increased IFNAR2 protein levels in B cells and T cells from carriers of the MS-protective class I alleles. (A) Correlation between rs2735099 and rs17199328 genotypes and imputed HLA-A (*n* = 301) and HLA-B alleles (*n* = 303). (B) The proportion of B cells subsets in bulk B cells (*n* = 95). (C) IFNAR2 surface levels in subsets of B cells from healthy donors (*n* = 95). (D) IFNAR2 surface levels in subsets of B cells and T cells from healthy donors (*n* = 22) and PwMS (*n* = 26). (B-D) Data are stratified by the sum of the MS-protective class I alleles HLA-A*02, HLA-A*68 and HLA-B*44, as indicated. p-values from the full model (B-C) or a simple linear regression (D), using the sum of MS-protective alleles (0–3) as a continuous variable. Boxplots show median, IQR and range. gMFI = geometric mean fluorescence intensity.

Imputation of HLA-A and HLA-B alleles from genotype data showed that the minor allele of rs2735099 tagged the HLA-A*02 and HLA-A*68 alleles, whereas rs17199328 tagged the HLA-B*44 allele (Fig 4A). Notably, both A*02 and B*44 are protective for multiple sclerosis (MS) [28–30] and a MS-protective effect of HLA-A*68 has also been suggested [28]. Consequently, these three alleles will hereafter be referred to as the as the MS-protective class I alleles.

Sub-phenotyping of B cells from 95 individuals revealed that the altered IFNAR2 levels were not due to differences in the proportion of B cell subsets (Fig 4B), but instead driven mainly by differential IFNAR2 surface levels in the IgD^–^CD27^+^ memory and IgD^–^CD27^–^ double-negative (DN) B cell subsets (p_memory_ = 5E-14, p_DN_ = 2E-10; Fig 4C). In T cells, the effect of the MS-protective class I alleles was evident in both naïve and memory subsets (S5B Fig). The increased IFNAR2 levels in B cells carrying MS-protective class I alleles was replicated in an independent set of 22 healthy individuals and 26 persons with MS (PwMS; Fig 4D).

In summary, we identify two additive pQTLs resulting in increased IFNAR2 surface levels in B and T cells carrying the MS-protective class I alleles A*02, A*68 and B*44.

### Post-transcriptional mechanisms underlie the increased IFNAR2 protein levels in carriers of the MS-protective class I alleles

**Fig 5:**
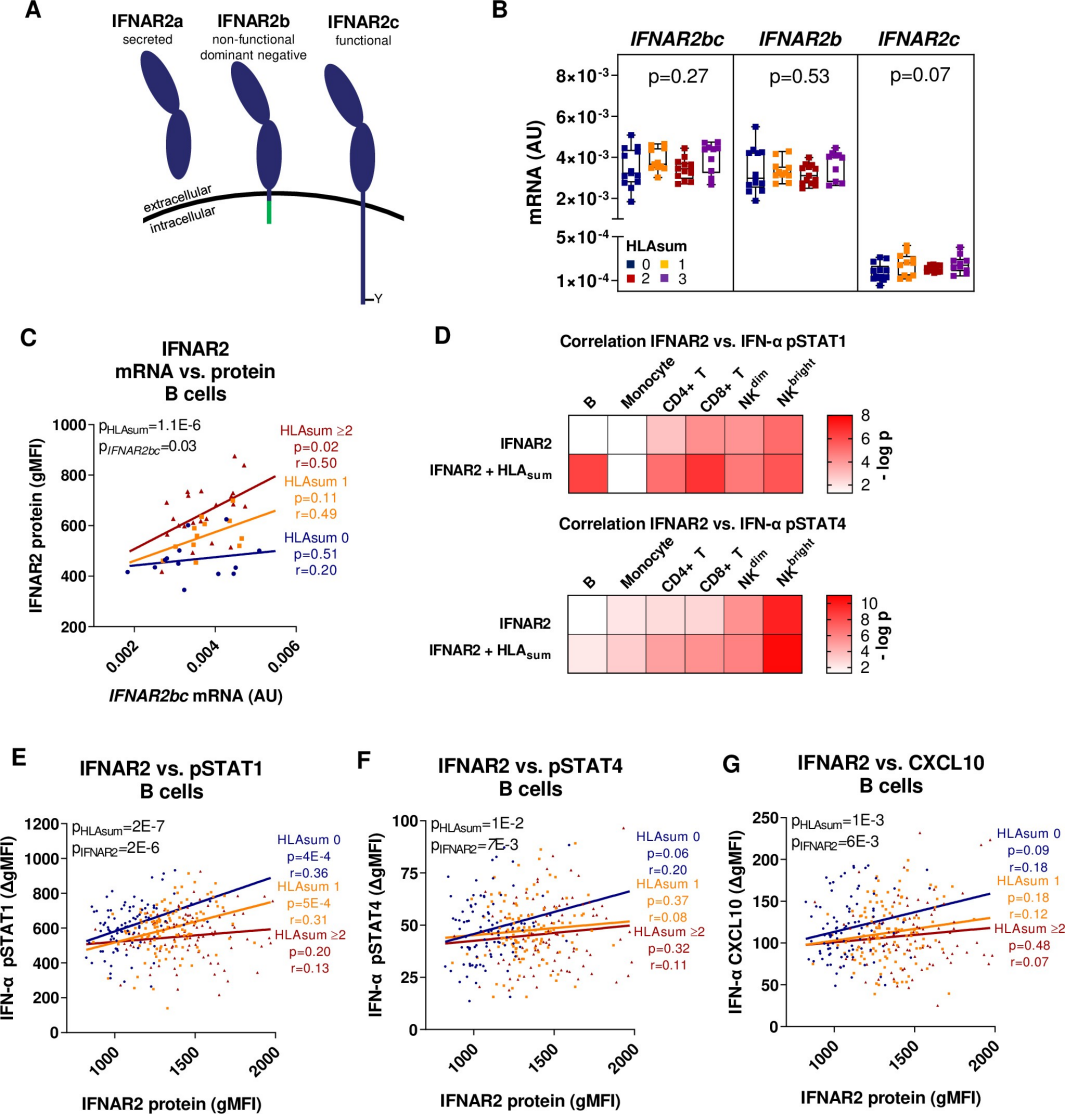
Decreased type I IFN response in B cells carrying the MS-protective class I alleles. (A) A schematic picture of the IFNAR2 isoforms. (B) *IFNAR2bc*, *IFNAR2b* and *IFNAR2c* mRNA levels in isolated B cells determined using qRT-PCR. mRNA levels are normalized to the expression of the reference gene *RPL13A* and expressed as arbitrary units (AU) (*n* = 47, simple linear regression using the sum of MS-protective class I alleles (HLAsum) as independent variable). (C) Correlation between *IFNAR2bc* mRNA levels and IFNAR2 surface receptor levels determined using flow cytometry (*n* = 47). Data are binned into 0, 1 and ≥2 HLAsum. p-values and Pearson’s correlation coefficient (r) from linear regressions of each group are shown in coloured text. p-values from multiple linear regressions of the combined data using HLAsum (continuous variable) and mRNA values as independent variables in the full model are denoted in black. (D) Heat-maps comparing the correlation between IFNAR2 levels and IFN-α induced pSTAT1 (top) or pSTAT4 (bottom) in indicated cell-types with and without including HLAsum as a covariate in the full model (*n* = 303). (E-G) Correlation between IFNAR2 protein levels and IFN-α (2000 IU/ml) induced pSTAT1 (E), pSTAT4 (F) and CXCL10 (G) in B cells. p-values as described in (C), (*n* = 303). Boxplots show median, IQR and range. gMFI = geometric mean fluorescence intensity.

IFNAR2 is expressed as a soluble secreted isoform (IFNAR2a) and two membrane-bound isoforms consisting of a full-length signalling receptor (IFNAR2c) and a truncated non-signalling receptor (IFNAR2b), which only differ in their intra-cellular domains (Fig 5A) [31]. The anti-IFNAR2 mAb used in our initial pQTL analysis targets the extracellular part of IFNAR2 and thus detects both membrane-bound IFNAR2 isoforms. To study whether the association between the MS-protective class I alleles and IFNAR2 protein levels was due to altered transcription, IFNAR2 mRNA expression levels were determined in B cells (*n* = 47) using primers that either amplify both membrane-bound isoforms (*IFNAR2bc*), or primers specific for the *IFNAR2b* or *IFNAR2c* isoforms. There were no significant differences in total mRNA levels of *IFNAR2* (*IFNAR2bc*), nor its isoforms, attributed to the MS-protective class I alleles (Fig 5B). The lack of association was confirmed in RNA expression microarray data on B cells [32] (*n* = 288; p > 0.10 for the three probes targeting IFNAR2) and RNA-sequencing (RNA-seq) data on lymphoblastoid cell lines (LCLs) [33] (*n* = 373 of European descent; p > 0.10 for 8 different IFNAR2 transcripts).

Correlation analysis between mRNA and protein levels revealed that while *IFNAR2bc* mRNA levels correlated with IFNAR2 protein levels in carriers of ≥2 MS-protective class I alleles, such correlation was not present in individuals carrying 0 MS-protective class I alleles (Fig 5C).

In summary, these data demonstrate that post-transcriptional mechanisms underlie the association between IFNAR2 protein levels and the MS-associated class I alleles.

### Decreased response to type I IFN stimulation in B cells and T cells carrying the MS-protective class I alleles

In order to study the downstream consequences of altered IFNAR2 protein levels, we evaluated the level of STAT phosphorylation after stimulation with IFN-α. Intriguingly, IFNAR2 levels did not correlate with IFN-α-induced pSTAT1 or pSTAT4 in B cells unless adjusting for the number of MS-protective class I alleles (Fig 5D), thereby demonstrating an effect of the MS-protective class I alleles on downstream IFNAR2 signalling. IFNAR2 levels correlated positively with IFN-α-induced pSTAT1 and pSTAT4 in B cells from individuals with 0 MS-protective class I alleles, but not in in B cells from carriers of ≥2 MS-protective class I alleles who had reduced STAT phosphorylation in response to IFN-α despite having higher IFNAR2 levels (Fig 5E and 5F). Downstream of the impaired type I IFN-induced STAT phosphorylaton, a decreased production of CXCL10 was observed in B cells carrying the MS-protective class I alleles (Fig 5G). Similar to B cells, both CD8^+^ T cells and CD4^+^ T cells carrying the MS-protective class I alleles displayed a decreased pSTAT1 and pSTAT4 in response to IFN-α (S6 Fig).

In an effort to investigate whether the reduced IFNAR response in carriers of the MS-protective class I alleles was due to increased levels of the dominant negative IFNAR2b isoform, we raised polyclonal anti-IFNAR2b and anti-IFNAR2c antibodies in rabbits. However, none of these antibodies, nor a commercial anti-IFNAR2c antibody, detected endogenous levels of IFNAR2 in primary cells.

In summary, these data for the first time link the MS-protective class I alleles to a decreased type I IFN response in B cells and T cells.

### The MS-protective class I alleles are *cis*-eQTLs for *HLA-J*

**Fig 6:**
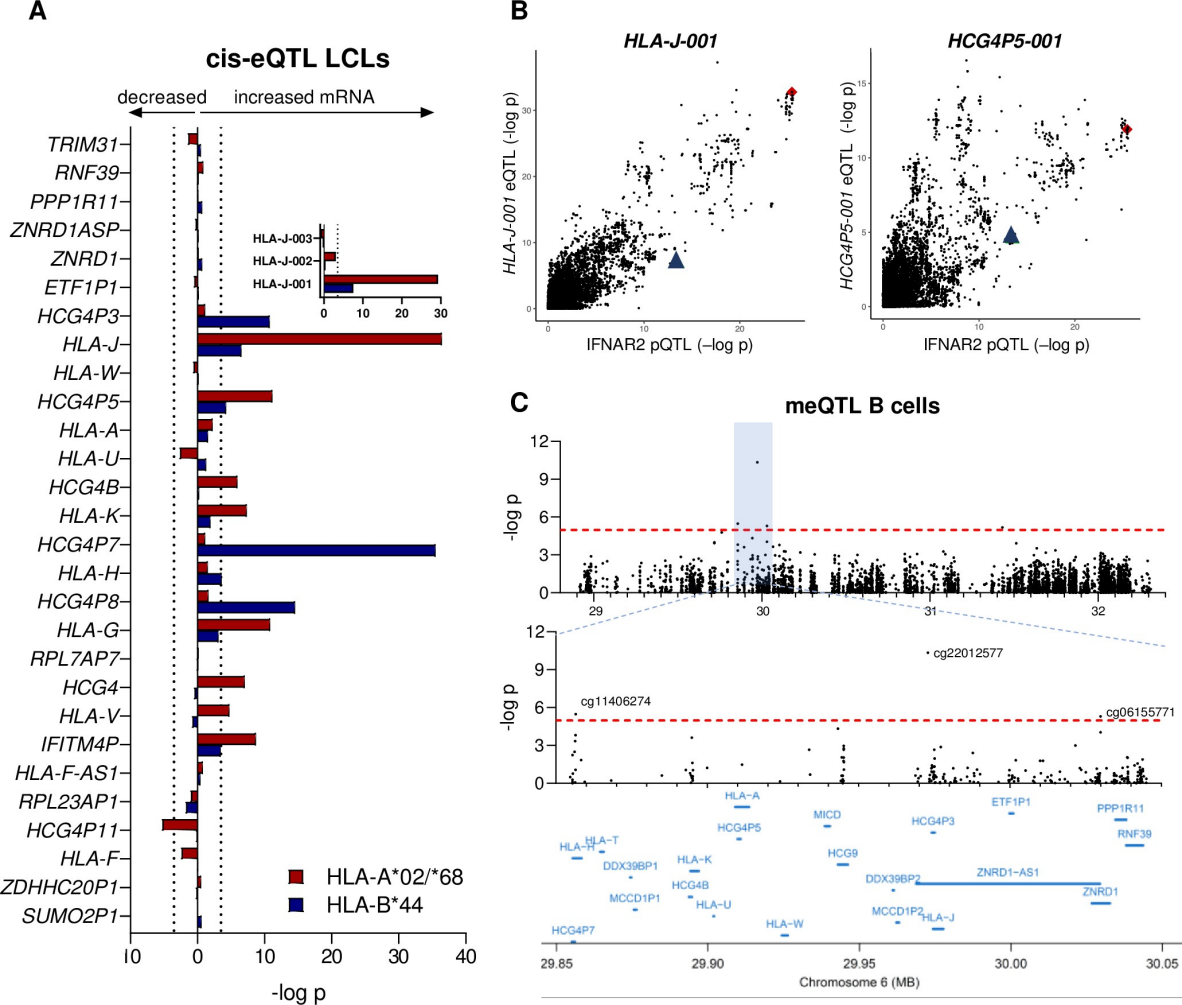
The MS-protective class I alleles are *cis*-eQTLs of *HLA-J*. (A) *Cis*-eQTL analysis within ± 1 Mb from the HLA-A*02/A*68 and HLA-B*44 tag SNPs in LCLs (*n* = 373). The p-value threshold of p < 3.2E-4, corresponding to a Bonferroni adjusted p-value of < 0.05 (155 analysed genes) is denoted with dotted lines. Data for 28 of the 155 genes are shown. The inset shows the transcript specific *cis*-eQTL p-values of *HLA-J*. (B) Correlation between p-values from the IFNAR2 pQTL in B cells and the *cis*-eQTL in LCLs for the transcript *HLA-J-001* (left) and *HCG4P5-001* (right). The HLA-A*02/A*68 and the B*44 tagging SNPs are marked in red and blue respectively. (C) *Cis*-meQTL analysis of the HLA-A*02/A*68 tagging SNP rs2735099 in primary B cells (*n* = 43). The p-value threshold of p < 2.1E-6, corresponding to a Bonferroni adjusted p-value of < 0.05 (23, 722 analysed CpG-sites) is denoted with dotted red lines.

As *trans*-pQTLs are commonly due to secondary effects from a *cis*-eQTL regulating protein expression of an intermediary protein [34], we next analyzed RNA-seq data from LCLs [33] for *cis*-eQTLs of the MS-protective class I alleles within a region extending 1 Mb from the two SNPs tagging these alleles. Assuming that the additive effect on IFNAR2 expression of HLA-A*02/A*68 and B*44 was driven by the same mechanisms, we searched for eQTLs with independent additive effects from the class I alleles. Using these criteria, we identified increased expression of the two pseudogenes *HLA-J* and *HCG4P5* in LCLs carrying the MS-protective class I alleles (p < 3.2E-4, corresponding to a p-value of < 0.05 after Bonferroni correction for 155 genes analysed in *cis*; Fig 6A). The *HLA-J* RNA sequencing data mapped to three different transcripts. The eQTL was specific for the HLA-J-001 (ENST00000494367.1) transcript (Fig 6A inset) and the eQTL association signal peak overlapped with the IFNAR2 pQTL peak (Fig 6B). *HCG4P5* has only one transcript, HCG4P5-001 (ENST00000429656.1) and the eQTL and pQTL association signals did not colocalize (Fig 6B). To search for co-regulated genes in *trans*, we performed a global eQTL analysis, but no *trans*-eQTLs were observed (p < 2.1E-6, corresponding to a p-value of 0.05 after Bonferroni correction for 23,722 analyzed genes).

As differential DNA methylation in transcriptional regulatory region is one possible mechanism driving eQTLs, we next analysed DNA methylation data from primary B cells [35]. This meQTL analysis identified a strong correlation between HLA-A*02/A*68 carriership and decreased methylation levels of a CpG-site 1 kb upstream of *HLA-J* (cg22012577, p = 4.6E-11; Fig 6C).

Together these data suggest that meQTL-mediated transcriptional regulation of *HLA-J* is a possible candidate mechanism to further explore in order to delineate the molecular mechanisms underlying the increased IFNAR2 protein levels and/or the uncoupling of IFNAR2 levels from type I IFN activation in carriers of the MS-protective class I alleles.

## Discussion

In this study we performed an extensive characterization of the genetic regulation of the type I and type II IFN receptor levels and their response in immune cells through genome-wide pQTL analysis. We identify several SNPs regulating IFN receptor levels and their responses in a cell-type specific manner. Our most important finding is the identification of two SNPs tagging the HLA-A*02/A*68 and the HLA-B*44 alleles that are independently and additively associated with increased surface levels of the IFNAR2 subunit in B cells and T cells. Notably, HLA-A*02 and HLA-B*44 are both protective for the development of MS [28–30], a chronic inflammatory disease of the central nervous system driven by the interaction of genetic and environmental factors [36]. Through its close relatedness to HLA-A*02, HLA-A*68 has been suggested to carry the same protective effect on MS as A*02 [28]. Protective effects of the infrequent HLA-B*38 and HLA-B*55 alleles have also been demonstrated [28–30]. Although we did not detect an effect of HLA-B*38 and HLA-B*55 on IFNAR2 levels in our study, firm conclusions cannot be drawn as the number of individuals carrying these alleles were low.

HLA-A*02 is beside HLA-DRB1*15:01 the strongest genetic association in MS, but the causal mechanisms underlying the protective function for MS is still unknown. Conceivably, such mechanisms may both include allelic differences in antigen presentation to CD8^+^ T cells, which may have both pathogenic and protective roles in MS or its experimental models [37–40], or effects unrelated to antigen presentation through regulation of mRNA expression of genes in the *HLA* locus [41].

The association between IFNAR2 levels and the MS-protective class I alleles was most prominent in B cells, and in particularly in the memory and double-negative subsets of B cells. Increased IFNAR2 levels were also seen in these B cell subsets from PwMS carrying the MS-protective class I alleles, but not in T cells. Although the absence of an effect in T cells from PwMS might reflect a power issue, these data confirm that the effect on IFNAR2 protein levels of the MS class I alleles are stronger in B cells than T cells. While classically considered a T cell driven disease, the importance of B cells in MS pathology is now well established, not least by the fact that anti-CD20 mAb is an effective treatment [42]. Both memory and double-negative B cells provide a link between the periphery and CNS as peripheral blood memory and double-negative B cells are clonally related to B cells found in the CSF of PwMS [43]. Memory B cells are of particular interest in MS as Epstein-Barr virus, which is a strong environmental risk factor for MS, reside in these cells. Moreover, the autoproliferation of brain-homing potentially pathogenic effector T cells is dependent on memory B cells [44], and depletion of memory B cells has been suggested as a possible mechanism of action for several of the therapies used today including IFN-β [45], anti-CD20 mAb, cladribine and natalizumab [46].

While *cis*-pQTLs are mainly driven by transcriptional regulation of the corresponding gene, *trans*-pQTLs are rarely directly connected to alteration in mRNA expression of the corresponding gene, but are rather driven by transcriptional regulation of a causal intermediate protein in *cis* of the pQTL [34]. Consistent with this observation, the increased protein levels of IFNAR2 in B cells from carriers of the MS-protective class I alleles were not under transcriptional control. Instead, we found that IFNAR2 protein levels were uncoupled from mRNA abundance in carriers lacking the MS-protective class I alleles.

Data from functional studies demonstrate that, despite presenting with higher IFNAR2 levels, B cells carrying the MS-protective class I alleles have a reduced pSTAT1, pSTAT4 and production of CXCL10 in response to type I IFN stimulation. CXCL10 is a potential biomarker for MS as the levels are elevated in cerebrospinal fluid (CSF) from PwMS and correlate with clinical characteristics such as IgG index, CSF mononuclear counts and T2 lesions [47, 48]. A decreased type I IFN response was also evident in CD4^+^ T and CD8^+^ T cells carrying the MS-protective class I alleles, but these effects were not as strong as in B cells. Although speculative, these data would fit in a model where the increased IFNAR2 levels in carriers of the MS-protective class I alleles are due to increased levels of the dominant inhibitory isoform IFNAR2b. The lack of isoform-specific anti-IFNAR2 antibodies precluded studies to directly determine whether this is true, and it is thus possible that other mechanisms, such as alterations in receptor trafficking/sorting or negative feedback signalling, are affected [49, 50].

Given the therapeutic use of IFN-β in MS, the association of decreased type I IFN activation in B cells and T cells carrying the MS-protective class I alleles may at first sound counter-intuitive. However, our data are in agreement with the fact that primary human immune cells carrying the MS-protective *TYK2* variant rs34536443 (Pro1104Ala) have a reduced response to IFN-β stimulation [51, 52] and that mice homozygous for the corresponding mutation (Pro1124Ala) are completely protected from developing the animal model of MS, experimental autoimmune encephalomyelitis (EAE) [52]. The protective effects of reduced type I IFN signalling in carriers of the MS protective *TYK2* variant might be driven by a shift between Th1/Th17 and Th2 polarization of T cells [51, 52]. A pathogenic role of type I IFN in the initiation of the MS disease is supported by the fact that antibody-mediated neutralization of type I IFN in the EAE model leads to a less severe disease during the early, but not in the established, phase of the disease [53]. Type I IFNs also have protective effects in EAE as both IFN-β and IFNAR1 knock-out mice have an increased disease severity [54, 55], but this effect is confined to the myeloid cells [54]. Thus, the effects of type I IFNs are complex and the effects on MS pathology probably differ depending on the cell-type and phase of the disease and whether a complete or partial reduction of IFN signalling is studied.

In an effort to determine the underlying molecular mechanism of the altered IFNAR2 levels we identified the IFN-α-induced pseudogene *HLA-J* [56] as a *cis*-eQTL for both MS-protective class I alleles. The differential expression of *HLA-J* was possibly mediated via altered DNA methylation as a meQTL upstream of *HLA-J* was observed for the HLA-A*02/*68 alleles. Although the term pseudogene implicates the absence of a functional effect, there is emerging data demonstrating biological effects of pseudogenes, including the regulation of immune responses during inflammation and infection [57–59]. Allelic variants of *HLA-J* have previously been shown to influence HLA-A surface protein levels [60] and it is possible that the same mechanisms operate for IFNAR2. However, further studies are needed in order to determine whether *HLA-J* mRNA levels are causatively linked to IFNAR2 protein levels, or whether these two intermediate phenotypes of the MS-protective class I alleles are epiphenomenally related.

Although a definite functional link between MS pathology and the observed differences in type I IFN response, in particular in B cells remains to be established, our data challenge the prevailing view that the protective influence of HLA-A*02 and B*44 are mechanistically related to preferences in peptide binding [61], either in the defence against a pathogen associated with MS such as EBV [62], or presentation of peptides related to CD8^+^ T cells involved in suppressing disease [39]. Instead, we put forward strong and unexpected evidence that their influence on MS is related to the type I interferon system. Further studies to unravel the detailed molecular mechanisms underlying the altered IFNAR2 protein levels and signalling in carriers of the MS-protective class I alleles are warranted as this may identify novel therapeutic targets to reduce the I IFN signalling specifically in B cells and T cells.

## Materials and methods

### Ethic statement

The study was approved by the Regional Ethical Review Board in Uppsala (2009/013) and Karolinska Institute (2009/2107-31/2). Written informed consent was obtained from all participants.

### Study participants

304 healthy individuals were recruited from Uppsala Bioresource. One individual was excluded during the genetic data quality control (see below) leaving a total of 303 healthy individuals. The median age was 52 years (range 22–81 years), 78% were females and 70% were CMV-seropositive. PBMCs were isolated from buffy coats using Ficoll density gradient centrifugation, and aliquots of 20E6 PBMCs were cryopreserved in FCS supplemented with 10% DMSO (Sigma-Aldrich). For replication, 22 additional healthy individuals from Uppsala Bioresource and 26 persons diagnosed with MS according to the revised McDonald criteria [63] were recruited. The median age of the PwMS was 41 years (range 28–63 years) and 85% were females. Of the PwMS, 24 presented with a relapsing-remitting MS in remission and two with a secondary-progressive MS. One of the PwMS was treatment naïve and 25 PwMS were treated with natalizumab. PBMCs were isolated from CPT-tubes and cryopreserved in FCS supplemented with 10% DMSO.

### Interferon stimulation of PBMCs

PBMCs were thawed in complete medium (RPMI-1640 (Sigma-Aldrich) supplemented with 2 mM HEPES, penicillin, streptomycin, L-glutamine and 10% FCS) and rested overnight in 6-well plates at 37°C in a cell incubator with 5% CO_2_. Cells were transferred to V-bottomed 96-well plates and fluorescently barcoded using CellTrace Violet (Invitrogen, 0 nM (unstimulated), 83 nM (IFN-α) and 667 nM (IFN-γ)), and subsequently cultured in the presence or absence of IFN-α2b (IntronA, MSD) or IFN-γ (PeproTech) at ~EC100 concentrations (2000 U/ml and 1 ng/ml, respectively) [64]. IFNAR2 and IFNGR1 levels were determined before stimulation. Phosphorylation of STAT1 and STAT4 was determined after 20 min, intra-cellular levels of CXCL9 and CXCL10 after 7 h with golgiplug (BD Biosciences) added during the last 6 h, and HLA class I and HLA class II expression after 28 h.

For the genome-wide association analysis, PBMCs were analysed in four consecutive experiments, and cells from 16 individuals were included as internal controls in all four experiments to allow for batch normalisation.

### Flow cytometry

Cells that were analysed for pSTAT and CXCL were fixed in 2% paraformaldehyde (PFA, Electron Microscopy Sciences) before flow cytometric stainings, whereas cells analysed for IFNR and HLA were fixed in 2% PFA after the last staining. Barcoded cells from each stimulus were pooled before flow cytometric stainings. For analysis of pSTAT and CXCL, cells were permeabilized with Perm Buffer III (BD Biosciences) or 0.5% saponin (Sigma-Aldrich), respectively. Stainings were performed in PBS supplemented with 2 mM EDTA and 0.5% human serum albumin (Octapharma) and FcBlock (Miltenyi Biotec) using two different 8-color panels with fluorochrome-conjugated antibodies (see below) and gating schemes as specified in S7 Fig. The IFNAR2 mAb REA124 was validated by transfection of HEK293T cells with siRNA targeting IFNAR2 ((dTdT-)GCACCATAGTGACACTGAA-dTdT), and plasmids encoding IFNAR2b or IFNAR2c (#RC201212 and #RC238664, Origene) (S8 Fig).

Flow cytometry data were acquired on a BD FACSCanto II instrument (BD Biosciences) and analysed with FlowJo version 10.5.3. IFN-induced read-outs were determined by subtracting the geometric mean fluorescence intensity (gMFI) in unstimulated cells from stimulated cells, i.e. ΔgMFI = gMFI_IFN_−gMFI_unstimulated_.

### Antibodies used in flow cytometry

#### Mouse monoclonal antibodies

CD3, REA613, APC-Vio770, Miltenyi Biotec, Cat# 130-113-136; RRID:AB_2725964

CD3, UCHT1, PerCP-Cy5.5, BD Biosciences, Cat# 560835; RRID:AB_2033956

CD4, SK3, BV510 BD Biosciences, Cat# 562970; RRID:AB_2744424

CD8, SK1, FITC, BD Biosciences, Cat# 345772

CD14, MφP9, FITC, BD Biosciences, Cat# 345784

CD16, 3G8, BV510, BD Biosciences, Cat# 563829; RRID:AB_2744296

CD19, HIB19, FITC, BD Biosciences, Cat# 555412; RRID:AB_395812

CD20, H1, PE-Cy7, BD Biosciences, Cat# 561175; RRID:AB_10562032

CD20, REA780, PE-Vio770, Miltenyi Biotec, Cat# 130-111-340; RRID:AB_2656074

CD27, M-T271, PE-Cy7, BD Biosciences, Cat# 560609; RRID:AB_1727456

CD45RA, HI100, APC-H7, BD Biosciences, Cat# 560674; RRID:AB_1727497

CD56, N901, PerCP-Cy5.5, Beckman Coulter, Cat# A79388

CD57, TBO1, PE-Cy7, eBiosciences, Cat# 25-0577-42; RRID:AB_2573354

IgD, IA6-2, APC-Cy7, Biolegend, Cat# 348218; RRID:AB_11203722

IFNAR2, REA124, APC, Miltenyi Biotec, Cat# 130-099-560; RRID:AB_2652223

IFNGR1, 92101, PE, R&D systems, Cat# FAB673P; RRID:AB_2264548

pSTAT1, 4a, AF647, BD Biosciences, Cat# 562070; RRID:AB_10896129

pSTAT4, 38-pSTAT4, BD Biosciences, Cat# 562073; RRID:AB_10895804

CXCL9, B8-11, PE, BD Biosciences, Cat# 566013; RRID:AB_2739458

CXCL10, REA334, APC, Miltenyi Biotec, Cat# 130-104-963; RRID:AB_2651478

HLA-ABC, REA230, PE, Miltenyi Biotec, Cat# 130-101-448; RRID:AB_2652082

HLA-DRPQ, REA332, APC, Miltenyi Biotec, Cat# 130-104-824; RRID:AB_2652180

#### Polyclonal antibodies

IFNAR1 rabbit polyclonal, Abgent, Cat# AP8550c; RRID:AB_10553443

IFNAR2c, rabbit polyclonal, Abcam, Cat# ab56070; RRID:AB_880736

IFNGR2, goat polyclonal, APC, R&D systems, Cat# FAB773A; RRID:AB_2121710

donkey polyclonal anti-Rabbit IgG, PE, BD Biosciences, Cat# 558416; RRID:AB_1645469

#### Genotyping, quality control and imputation of genetic variants

DNA from the healthy individuals were genotyped on the Infinium Global Screening Array v2.0 (Illumina). The genetic structure of the study population was analysed using EIGENSOFT [65] after excluding long-range linkage disequilibrium (LD) regions [66], SNVs with MAF < 0.05 and SNVs in LD r^2^ > 0.2 using the HapMap3 cohort as reference population. All individuals mapped to the CEU population (< 10 SD from the mean HapMAP CEU population for the first 5 principle components) and passed the exclusion criteria for heterozygosity (> 5 SD from population mean), call rate (< 98%) and discordance for reported sex. One individual was excluded due to relatedness (π-hat > 0.1875, PLINK software v1.90).

Variants with a call rate < 98%, a MAF < 1% or with deviation from Hardy-Weinberg equilibrium (p < 0.00001) were excluded. Imputation of additional variants was performed at the Sanger imputation service [67] using the Haplotype reference consortium r1.1 reference and the “pre-phase with EAGLE2 and impute” pipeline [68]. Imputed genotype calls with a genotype probability score below 0.9 were set to missing and variants with an info score below 0.8, a MAF < 0.01, deviation from Hardy-Weinberg equilibrium (p < 0.0001) or a call rate < 95% were excluded. The final dataset contained 303 individuals and 5,084,123 genetic variants.

#### Imputation of HLA alleles

HLA types were imputed using HIBAG version 1.18.1 [69] and the multi-ethnic prediction model specific for Illumina Infinium Global Screening Array v2.0.

#### CMV status

CMV status was determined serologically at the first visit at the blood donation centre. For 38 donors with missing information, CMV status was imputed from 270 flow cytometry-based traits (cell proportions and gMFI levels in the pQTL screening) using missForest version 1.4 [70]. The random forest-based method showed an average prediction accuracy of 86% in 100 trails in which 10% of individuals with known CMV status were randomly left out for imputation.

#### pQTL mapping

Flow cytometry data were normalised for inter-experimental variation by applying factor correction, i.e. the average of internal control samples for the individual experiment divided by the average of internal control samples for all four experiments. Each trait was assessed for normality by Shapiro–Wilk test and visual evaluation of Q-Q plots. Data that were not normally distributed were square root or log-transformed. The pQTL analysis was performed by linear regression using PLINK version 1.90b6.7 [71]. Age, sex, CMV status, experiment batch, year and season (summer/winter) of blood sampling were included as covariates and is referred to as the “full model”. For the genome-wide association analysis, the standard threshold of p < 5.0E-8 was applied, whereas for other analyses, a p-value < 0.05 was considered significant.

#### mRNA expression analysis

B cells and monocytes were isolated sequentially from 40E6 PBMCs from 48 healthy individuals through positive selection of CD19^+^ cells followed by positive selection for CD14^+^ cells (MACS Cell Separation, Miltenyi Biotec). RNA was isolated using NucleoSpin RNA kit (Macherey-Nagel), and cDNA generated using QuantiTect Reverse Transcription kit (Qiagen). B cell mRNA from one individual was excluded due to an A260/A280 ratio of 0.9. Fast TaqMan qPCR was performed using TaqMan Fast Advanced Master Mix (Thermo Fisher Scientific) and TaqMan gene expression assays: Hs01022064_m1 (*IFNAR2c*), Hs00174198_m1 (*IFNAR2bc)*, Hs00988304_m1 (*IFNGR1*), Hs03043885_g1 (*RPL13A*) (all from ThermoFischer). mRNA levels of IFNAR2b were determined with custom designed primers and probes [72]: Forward primer: CTATTCACAGGTGCAGTCATAATGC, Reverse primer:: GCACGCTTGTAATCCCAGCTA, Taqman probe: FAM-CAGTCGTCCTGCCTAAGCTTCCCCA-TAMRA. Data was acquired using a 7500 Fast Real-Time PCR System (Applied Biosystems) and analysed by the 2^-ΔCt^ method using *RPL13A* as the reference gene.

#### eQTL analysis of publically available datasets

Microarray data on IFNAR2 expression in B cells from 288 individuals [32] were analysed by linear regression adjusting for sex using the proxies rs2735099 > rs2735097 (R^2^_GBR_ = 0.88) and rs17199328 > rs2596477 (R^2^_GBR_ = 1.0). RNA-seq data on LCLs from 373 individuals of European descent [33] were analysed using a linear regression adjusting for sex and population using the proxy rs2735099 > rs2735097 (R^2^_EUR_ = 0.92).

#### meQTL analysis

Previously published data on CpG DNA methylation levels (HM450K BeadChip, Illumina) in B cells from 43 healthy individuals [35], were analysed for meQTLs within a 1 MB window of rs2735099 using a linear regression model including age and sex as covariates.

#### Statistical analysis

Statistical analyses were performed in R version 3.5.1 [73] or GraphPad Prism version 8. All statistical tests were two-tailed.

## Supporting information

S1 Fig Cell-type specific interferon receptor expression and response. (A) Basal levels of IFNAR2 (red) and IFNGR1 (blue) surface protein levels in indicated subsets of immune cells. (B-D) IFN-α (red) and IFN-γ-induced (blue) activation of cells measured as (B) phosphorylation of STAT1 (pSTAT1) and STAT4 (pSTAT4), (C) intra-cellular levels of CXCL9 and CXCL10 and (D) HLA-class I and HLA-class II surface protein levels in indicated subsets of immune cells. The geometric mean fluorescence intensity (gMFI) is presented as violin plots with median and interquartile range denoted. For the IFN-stimulated traits the gMFI from unstimulated cells has been subtracted.
(PDF)

S2 Fig A false positive pQTL signal resulting from differential Fc-receptor binding. (A) Regional association plot of the IFNGR1 surface levels in monocytes determined using the anti-IFNGR1 mAb clone 92101 of the IgG1 subtype. (B-C) Boxplots for monocyte flow cytometry stainings stratified by rs1801274 using anti-IFNGR1 mAb 92101 (B, n = 303) or REA161 which is recombinantly engineered to lack Fc-receptor bindings (C, n = 94). (D-E) Correlation between *IFNGR1* mRNA levels and IFNGR1 protein levels determined with clone REA161 in (D) or clone 92101 in (E) (n = 48). (F) Correlation between anti-IFNGR1 flow cytometry stainings with clone 92101 and REA161 with and without (all) stratification by rs1801274 (n = 94). (A-C) p-values from the full single SNP model. (D-F) p-values from simple linear regressions and Pearson’s correlation coefficient (r) is denoted. (B-C) Boxplots show median, IQR and range. gMFI = geometric mean fluorescence intensity.
(PDF)

S3 Fig Cell-type specificity of IFNAR2 cis-pQTLs. (A) Regional association plots and (B) boxplots for the IFNAR2 cis-pQTLs in indicated subsets of immune cells. p-values from the full model with a single SNP included in all figures except for CD56^bright^ NK cells panel in (B) where the two indicated SNPs were included in an additive model. Boxplots show median, IQR and range. gMFI = geometric mean fluorescence intensity.
(PDF)

S4 Fig Characterization of the IFN-α induced pSTAT/*FOCAD* pQTL. (A) Regional association plots of IFN-α induced phosphorylation of STAT4 (pSTAT4) or STAT1 (pSTAT1) in indicated subsets of immune cells. (B, D and E) Boxplots for IFN-α-induced pSTAT1/4 (B), CXCL9 (D) and CXCL10 (E), in indicated subsets of immune cells stratified for rs2298260. (C) Conditional analysis of IFN-α-induced pSTAT4 in CD56^dim^ NK cells after conditioning on rs7388989 (top) or rs2298260 (bottom). (A-E) p-values from the full single SNP model. Boxplots show median, IQR and range. gMFI = geometric mean fluorescence intensity.
(PDF)

S5 Fig Cell-type specificity for the IFNAR2/*HLA* pQTL. (A) Boxplots of IFNAR2 surface levels stratified by rs2735099 and rs17199328 in indicated subsets of immune cells. p-values from the full model with two additive SNPs. (B) Violin plots of IFNAR2 levels in subsets of CD8^+^ T cells and CD4^+^ T cells as specified. Individuals are stratified by the sum of HLA-A*02, A*68 and B*44 alleles. p-values from the full model using the sum of HLA-A*02, A*68 and B*44 (0–4) as a continuous variable.
(PDF)

S6 Fig Decreased type I IFN response in T cells carrying the MS-protective class I alleles. Correlation between IFNAR2 protein levels and IFN-α (2000 IU/ml) induced pSTAT1 or pSTAT4 in CD8^+^ and CD4^+^ T cells, as indicated. Data are binned into 0, 1 and ≥2 HLAsum. p-values and Pearson’s correlation coefficient (r) from linear regressions of each group are shown in coloured text. p-values from multiple linear regressions of the combined data using HLAsum (continuous variable) and IFNAR2 protein levels as independent variables in the full model are denoted in black (*n* = 303).
(PDF)

S7 Fig Gating strategy. Gating strategy for (A) interferon receptor (IFNR) panels, (B) STAT phosphorylation panels, (C) CXCL panels, (D) HLA panels, (E) B cell subsets, (B-D) Histograms are coloured according to stimuli: Medium (red), IFN-α (blue), IFN-γ (orange). DCM—dead cell marker.
(PDF)

S8 Fig Validation of the monoclonal IFNAR2 antibody REA124. HEK293T cells were transfected with (A) siRNA targeting a sequence shared between IFNAR2b and IFNAR2c ((dTdT-)GCACCATAGTGACACTGAA-dTdT), or (B) plasmids encoding myc-DKK-tagged IFNAR2b or IFNAR2c (#RC201212 and #RC238664, Origene). Cells were stained with anti-IFNAR2 mAb REA124, which recognize both IFNAR2b and IFNAR2c. Stainings for each condition are compared to non-transfected cells.
(PDF)

S1 Data An excel file containing raw data on geometric mean fluorescence intensities, covariates and HLA-A and HLA-B imputed alleles. (XLSX)

S2 Data An Excel file with p-values for all 45 traits for each SNP with p < 1E-4 for at least one trait in the pQTL mapping. (XLSX)

S3 Data An Excel file with data underlying figures. (XLSX)
