# Supplementary material for: Function of multiple sclerosis-protective HLA class I alleles revealed by genome-wide protein-quantitative trait loci mapping of interferon signalling
Source: PLoS Genet. 2020 Oct 26;16(10):e1009199. doi: 10.1371/journal.pgen.1009199 (PMC7644105; doi:10.1371/journal.pgen.1009199)
Supplement: S1 Fig — (A) Basal levels of IFNAR2 (red) and IFNGR1 (blue) surface protein levels in indicated subsets of immune cells. (B-D) IFN-α (red) and IFN-γ-induced (blue) activation of cells measured as (B) phosphorylation of STAT1 (pSTAT1) and STAT4 (pSTAT4), (C) intra-cellular levels of CXCL9 and CXCL10 and (D) HLA-class I and HLA-class II surface protein levels in indicated subsets of immune cells. The geometric mean fluorescence intensity (gMFI) is presented as violin plots with median and interquartile range denoted. For the IFN-stimulated traits the gMFI from unstimulated cells has been subtracted. (PDF) [file pgen.1009199.s001.pdf]

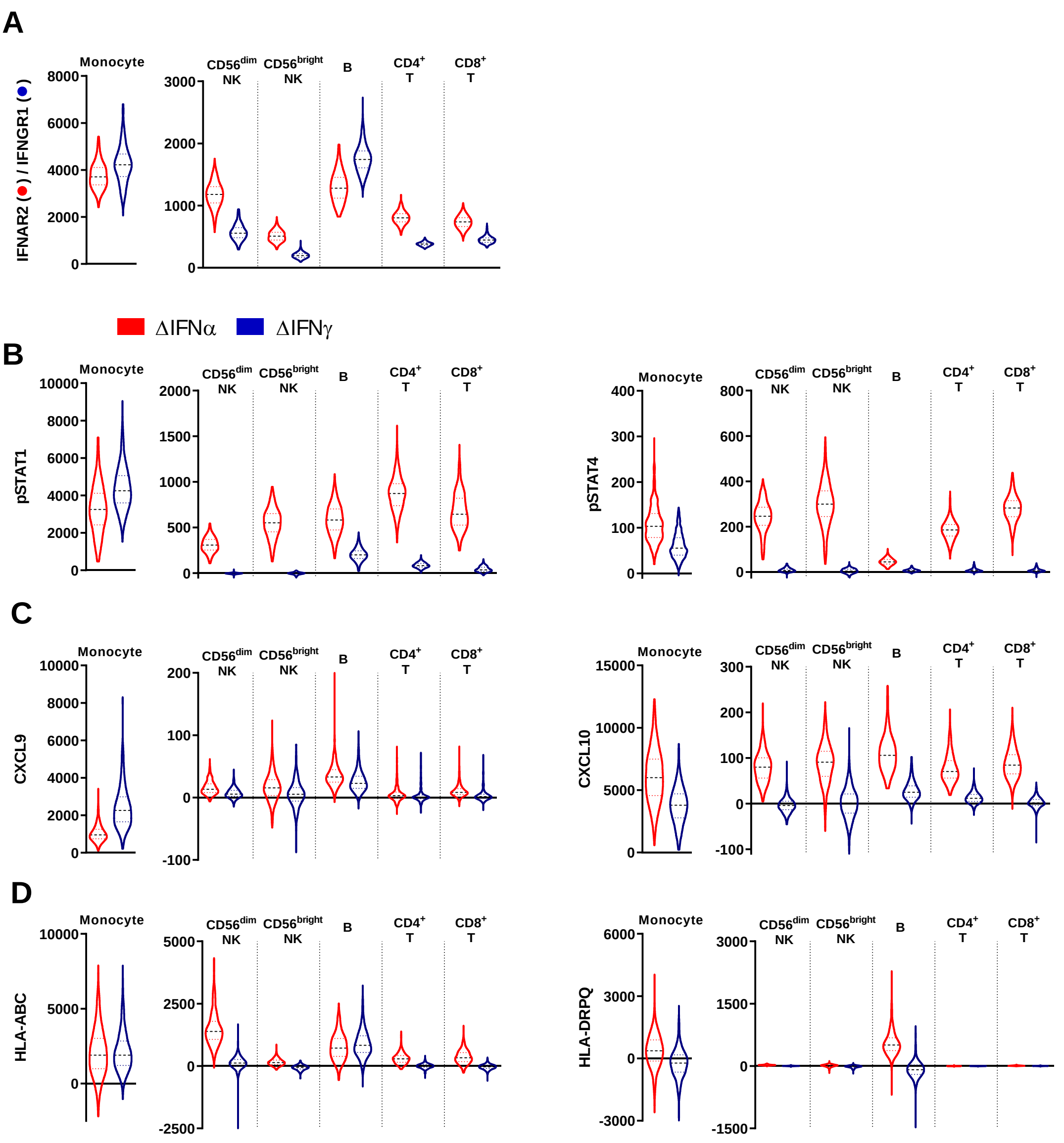

S1 Fig. Cell.-type specific interferon receptor expression and response. (A) Basal levels of IFNAR2 (red) and IFNGR1 (blue) surface protein levels in indicated subsets of immune cells. (B-D) IFN-α (red) and IFN-γ-induced (blue) activation of cells measured as (B) phosphorylation of STAT1 (pSTAT1) and STAT4 (pSTAT4), (C) intra-cellular levels of CXCL9 and CXCL10 and (D) HLA-class I and HLA-class II surface protein levels in indicated subsets of immune cells. The geometric mean fluorescence intensity (gMFI) is presented as violin plots with median and interquartile range denoted. For the IFN-stimulated traits the gMFI from unstimulated cells has been subtracted.
