# Supplementary material for: Function of multiple sclerosis-protective HLA class I alleles revealed by genome-wide protein-quantitative trait loci mapping of interferon signalling
Source: PLoS Genet. 2020 Oct 26;16(10):e1009199. doi: 10.1371/journal.pgen.1009199 (PMC7644105; doi:10.1371/journal.pgen.1009199)
Supplement: S2 Fig — (A) Regional association plot of the IFNGR1 surface levels in monocytes determined using the anti-IFNGR1 mAb clone 92101 of the IgG1 subtype. (B-C) Boxplots for monocyte flow cytometry stainings stratified by rs1801274 using anti-IFNGR1 mAb 92101 (B, n = 303) or REA161 which is recombinantly engineered to lack Fc-receptor bindings (C, n = 94). (D-E) Correlation between IFNGR1 mRNA levels and IFNGR1 protein levels determined with clone REA161 in (D) or clone 92101 in (E) (n = 48). (F) Correlation between anti-IFNGR1 flow cytometry stainings with clone 92101 and REA161 with and without (all) stratification by rs1801274 (n = 94). (A-C) p-values from the full single SNP model. (D-F) p-values from simple linear regressions and Pearson’s correlation coefficient (r) is denoted. (B-C) Boxplots show median, IQR and range. gMFI = geometric mean fluorescence intensity. (PDF) [file pgen.1009199.s002.pdf]

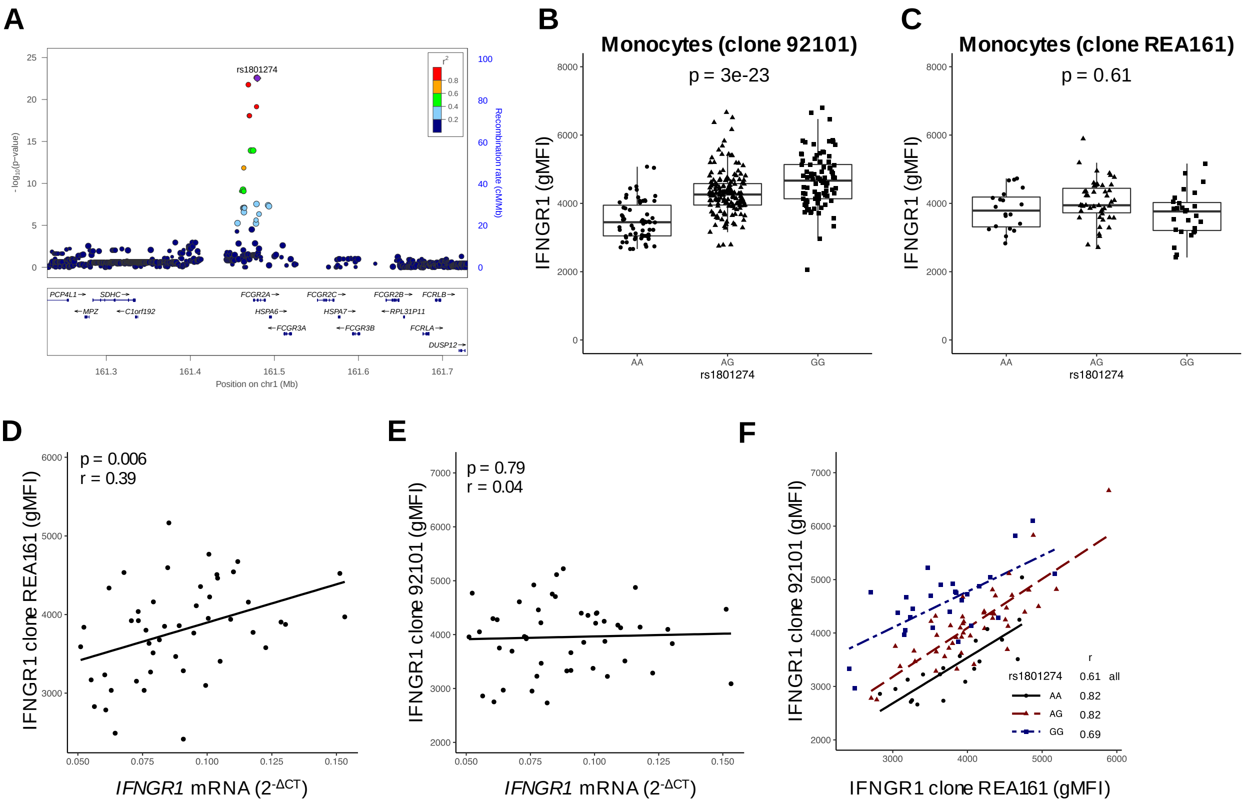

S2 Fig. A false positive pQTL signal resulting from differential Fc-receptor binding. (A) Regional association plot of IFNGR1 surface levels in monocytes determined using the anti-IFNGR1 mAb clone 92101 of the IgG1 subtype. (B-C) Boxplots for monocyte flow cytometry stainings stratified by rs1801274 using anti-IFNGR1 mAb 92101 (B, n=303) or REA161 (C, n=94), which is recombinantly engineered to lack Fc-receptor bindings. (D-E) Correlation between *IFNGR1* mRNA levels and IFNGR1 protein levels determined with clone REA161 in (D) or clone 92101 in (E) (n=48). (F) Correlation between anti-IFNGR1 flow cytometry stainings with clone 92101 and REA161 with and without (all) stratification by rs1801274 (n=94). (A-C) p-values from the full single SNP model. (D-F) p-values from simple linear regressions and Pearson's correlation coefficient (r) is denoted. (B-C) Boxplots show median, IQR and range. gMFI= geometric mean fluorescence intensity
