# Supplementary material for: Function of multiple sclerosis-protective HLA class I alleles revealed by genome-wide protein-quantitative trait loci mapping of interferon signalling
Source: PLoS Genet. 2020 Oct 26;16(10):e1009199. doi: 10.1371/journal.pgen.1009199 (PMC7644105; doi:10.1371/journal.pgen.1009199)
Supplement: S3 Fig — (A) Regional association plots and (B) boxplots for the IFNAR2 cis-pQTLs in indicated subsets of immune cells. p-values from the full model with a single SNP included in all figures except for CD56bright NK cells panel in (B) where the two indicated SNPs were included in an additive model. Boxplots show median, IQR and range. gMFI = geometric mean fluorescence intensity. (PDF) [file pgen.1009199.s003.pdf]

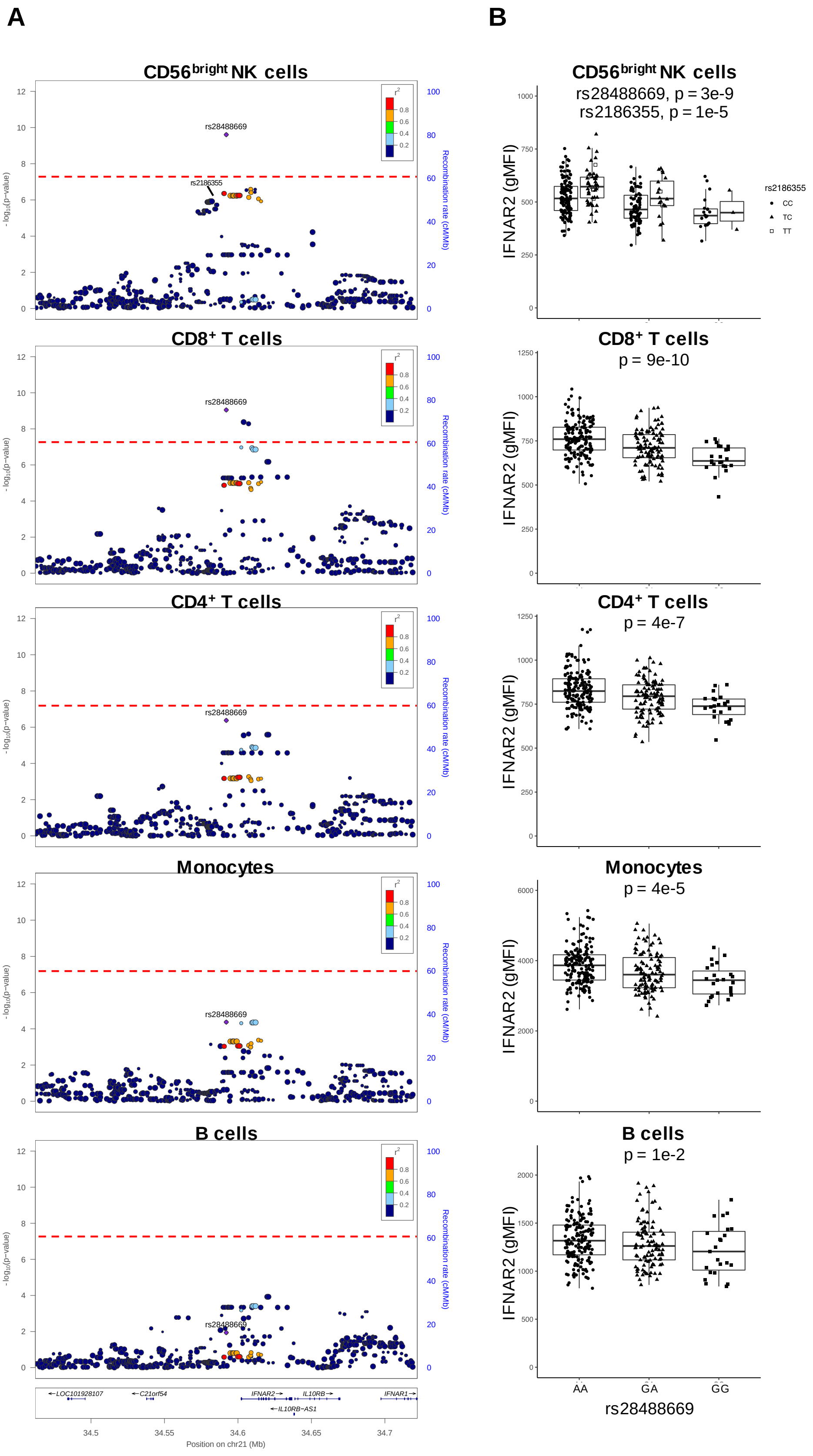

S3 Fig. Cell-type specificity of IFNAR2 cis-pQTLs. (A) Regional association plots and (B) boxplots for the IFNAR2 cis-pQTLs in indicated subsets of immune cells. p-values from the full model with a single SNP included in all figures except for CD56^{bright} NK cells panel in (B) where the two indicated SNPs were included in an additive model. Boxplots show median, IQR and range. gMFI= geometric mean fluorescence intensity
