# Supplementary material for: Function of multiple sclerosis-protective HLA class I alleles revealed by genome-wide protein-quantitative trait loci mapping of interferon signalling
Source: PLoS Genet. 2020 Oct 26;16(10):e1009199. doi: 10.1371/journal.pgen.1009199 (PMC7644105; doi:10.1371/journal.pgen.1009199)
Supplement: S4 Fig — (A) Regional association plots of IFN-α induced phosphorylation of STAT4 (pSTAT4) or STAT1 (pSTAT1) in indicated subsets of immune cells. (B, D and E) Boxplots for IFN-α-induced pSTAT1/4 (B), CXCL9 (D) and CXCL10 (E), in indicated subsets of immune cells stratified for rs2298260. (C) Conditional analysis of IFN-α-induced pSTAT4 in CD56dim NK cells after conditioning on rs7388989 (top) or rs2298260 (bottom). (A-E) p-values from the full single SNP model. Boxplots show median, IQR and range. gMFI = geometric mean fluorescence intensity. (PDF) [file pgen.1009199.s004.pdf]

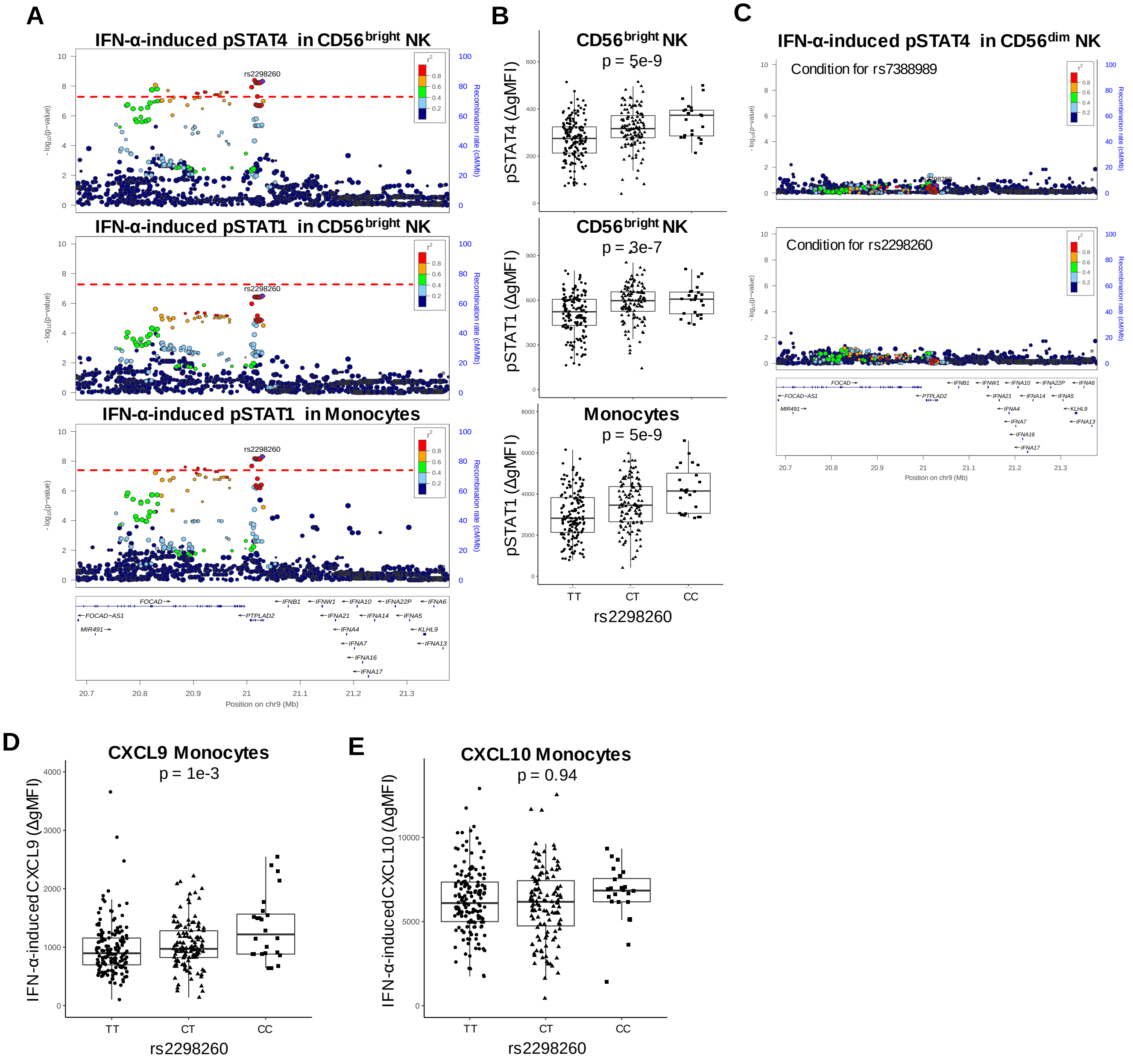

S4 Fig. Characterization of the IFN-α induced pSTAT/*FOCAD* pQTL. (A) Regional association plots of IFN-α induced phosphorylation of STAT4 (pSTAT4) or STAT1 (pSTAT1) in indicated subsets of immune cells. (B, D and E-D) Boxplots for IFN-α-induced pSTAT1/4 (B), CXCL9 (D) and CXCL10 in (E), in indicated subsets of immune cells stratified for rs2298260. (C) Conditional analysis of IFN-α-induced pSTAT4 in CD56^{dim} NK cells after conditioning on rs7388989 (top) or rs2298260 (bottom). (A-E) p-values from the full single SNP model. Boxplots show median, IQR and range. gMFI= geometric mean fluorescence intensity
