# Supplementary material for: Function of multiple sclerosis-protective HLA class I alleles revealed by genome-wide protein-quantitative trait loci mapping of interferon signalling
Source: PLoS Genet. 2020 Oct 26;16(10):e1009199. doi: 10.1371/journal.pgen.1009199 (PMC7644105; doi:10.1371/journal.pgen.1009199)
Supplement: S5 Fig — (A) Boxplots of IFNAR2 surface levels stratified by rs2735099 and rs17199328 in indicated subsets of immune cells. p-values from the full model with two additive SNPs. (B) Violin plots of IFNAR2 levels in subsets of CD8+ T cells and CD4+ T cells as specified. Individuals are stratified by the sum of HLA-A*02, A*68 and B*44 alleles. p-values from the full model using the sum of HLA-A*02, A*68 and B*44 (0–4) as a continuous variable. (PDF) [file pgen.1009199.s005.pdf]

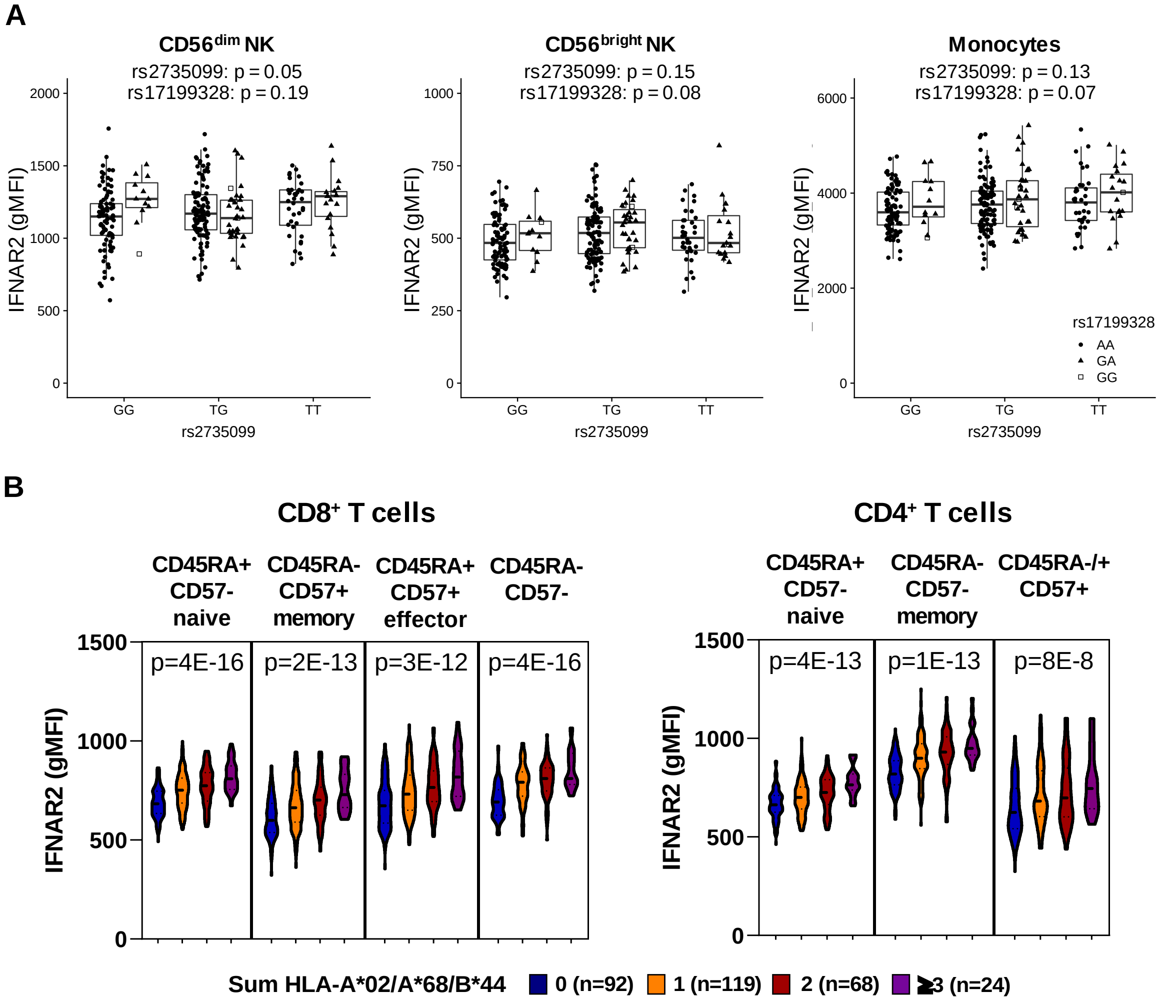

S5 Fig. Cell-type specificity for the IFNAR2/*HLA* pQTL. (A) Boxplots of IFNAR2 surface levels stratified by rs2735099 and rs17199328 in indicated subsets of immune cells. p-values from the full model with two additive SNPs. (B) Violin plots of IFNAR2 levels in subsets of $CD8^+$ T cells and $CD4^+$ T cells as specified. Individuals are stratified by the sum of HLA-A*02, A*68 and B*44 alleles. p-values from the full model using the sum of HLA-A*02, A*68 and B*44 (0-4) as a continuous variable.
