# Supplementary material for: Function of multiple sclerosis-protective HLA class I alleles revealed by genome-wide protein-quantitative trait loci mapping of interferon signalling
Source: PLoS Genet. 2020 Oct 26;16(10):e1009199. doi: 10.1371/journal.pgen.1009199 (PMC7644105; doi:10.1371/journal.pgen.1009199)
Supplement: S6 Fig — Correlation between IFNAR2 protein levels and IFN-α (2000 IU/ml) induced pSTAT1 or pSTAT4 in CD8+ and CD4+ T cells, as indicated. Data are binned into 0, 1 and ≥2 HLAsum. p-values and Pearson’s correlation coefficient (r) from linear regressions of each group are shown in coloured text. p-values from multiple linear regressions of the combined data using HLAsum (continuous variable) and IFNAR2 protein levels as independent variables in the full model are denoted in black (n = 303). (PDF) [file pgen.1009199.s006.pdf]

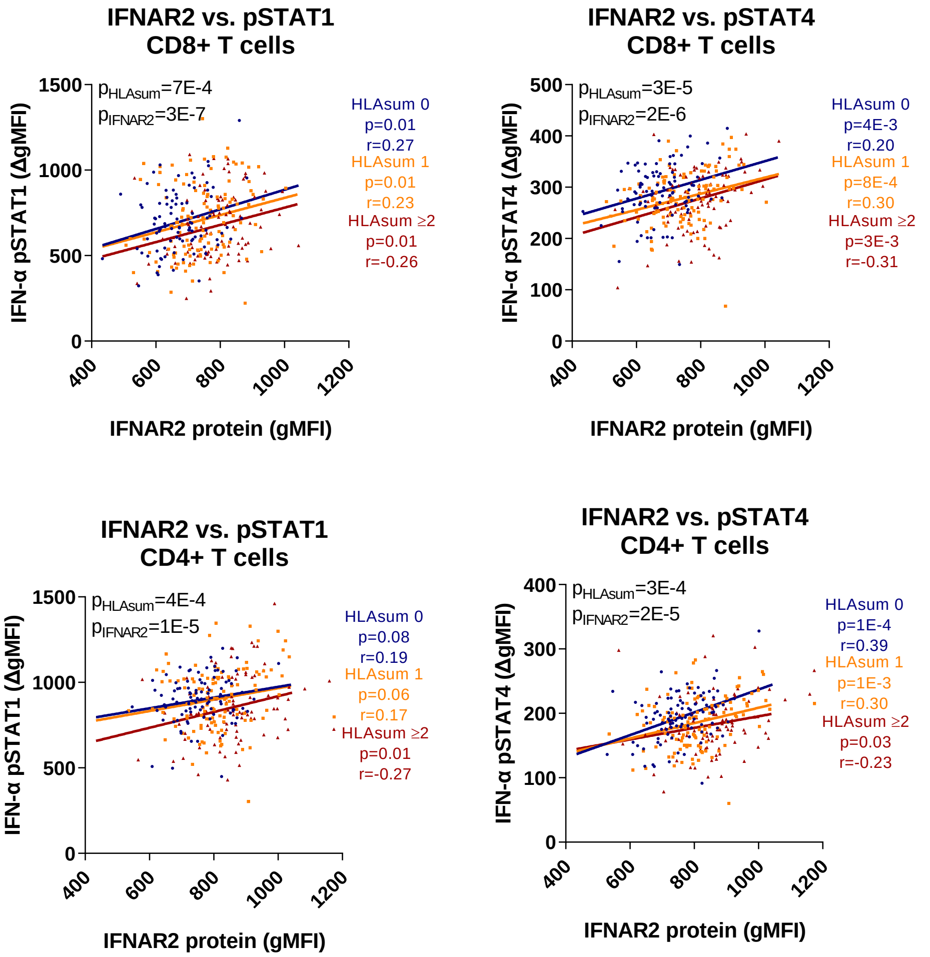

S6 Fig. Decreased type I IFN response in T cells carrying the MS-protective class I alleles.
Correlation between IFNAR2 protein levels and IFN-α (2000 IU/ml) induced pSTAT1 or pSTAT4 in $CD8^+$ and $CD4^+$ T cells, as indicated. Data are binned into 0, 1 and ≥2 HLAsum. p-values and Pearson's correlation coefficient (r) from linear regressions of each group are shown in coloured text. p-values from multiple linear regressions of the combined data using HLAsum (continuous variable) and IFNAR2 protein levels as independent variables in the full model are denoted in black (n = 303).
