# Supplementary material for: Function of multiple sclerosis-protective HLA class I alleles revealed by genome-wide protein-quantitative trait loci mapping of interferon signalling
Source: PLoS Genet. 2020 Oct 26;16(10):e1009199. doi: 10.1371/journal.pgen.1009199 (PMC7644105; doi:10.1371/journal.pgen.1009199)
Supplement: S7 Fig — Gating strategy for (A) interferon receptor (IFNR) panels, (B) STAT phosphorylation panels, (C) CXCL panels, (D) HLA panels, (E) B cell subsets, (B-D) Histograms are coloured according to stimuli: Medium (red), IFN-α (blue), IFN-γ (orange). DCM—dead cell marker. (PDF) [file pgen.1009199.s007.pdf]

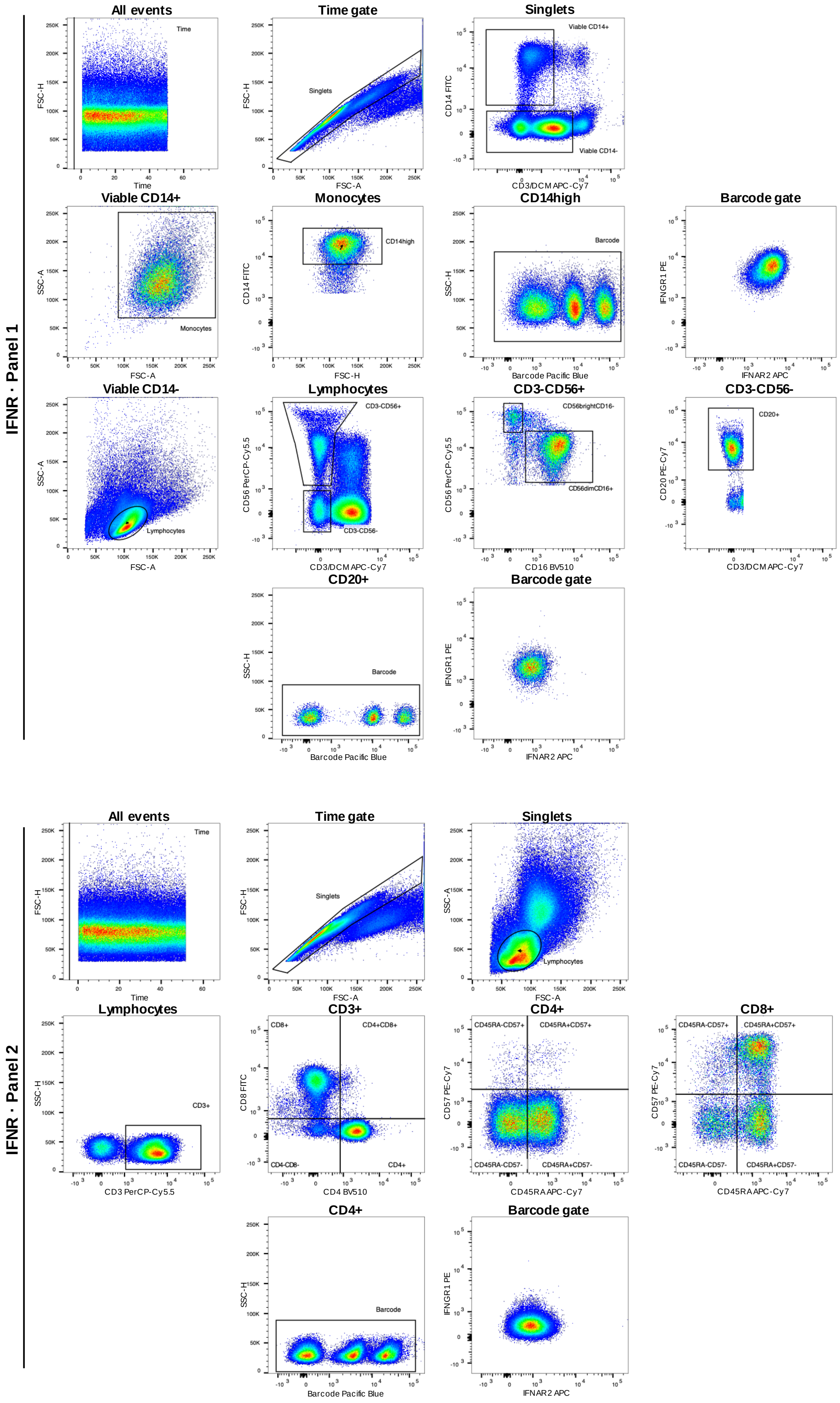

S7A Fig. Gating strategy for interferon receptor (IFNR) panels. DCM - dead cell marker

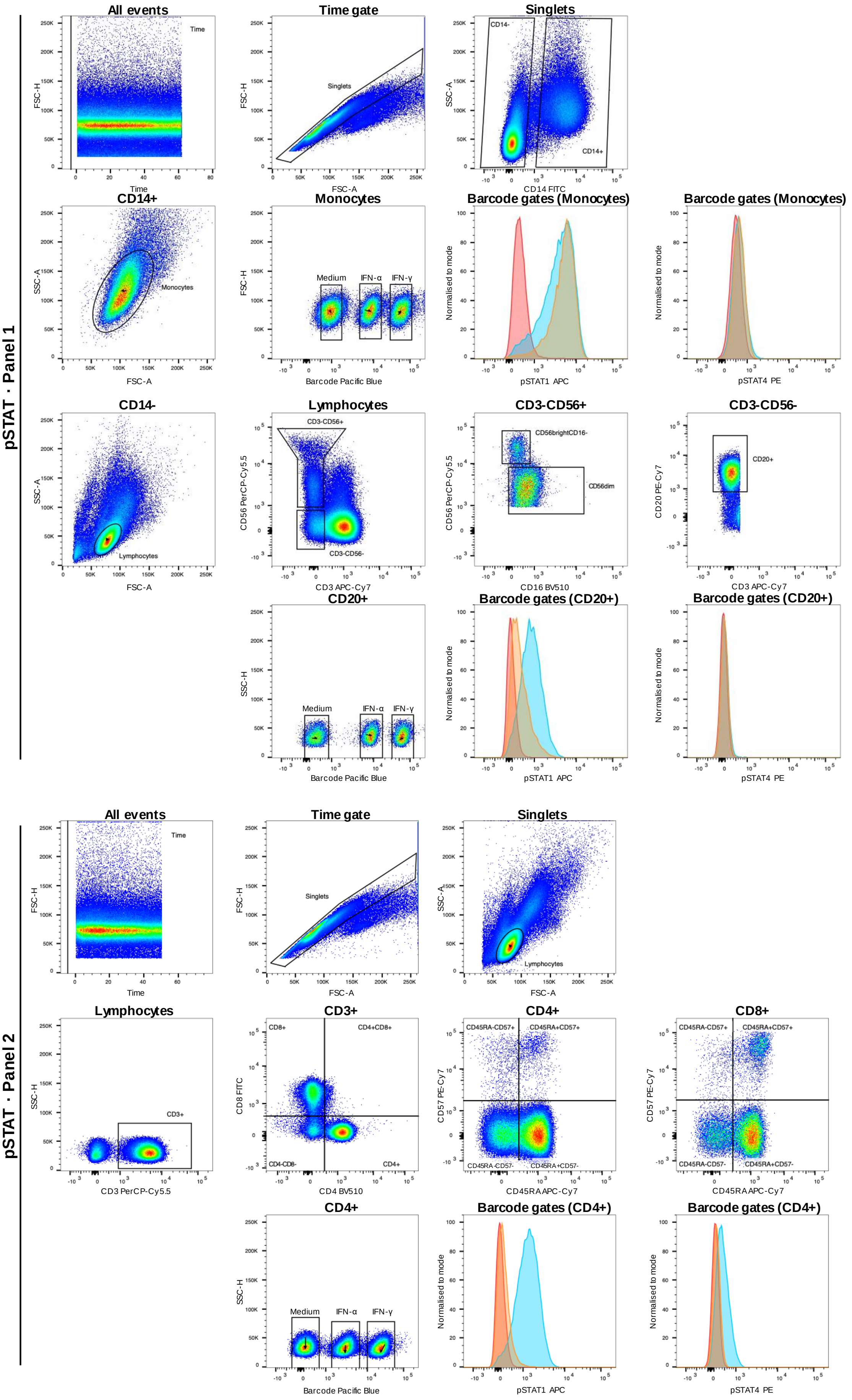

S7B Fig. Gating strategy for STAT phosphorylation panels. Histograms for pSTAT: Medium (red), IFN-α (blue), IFN-γ (orange).

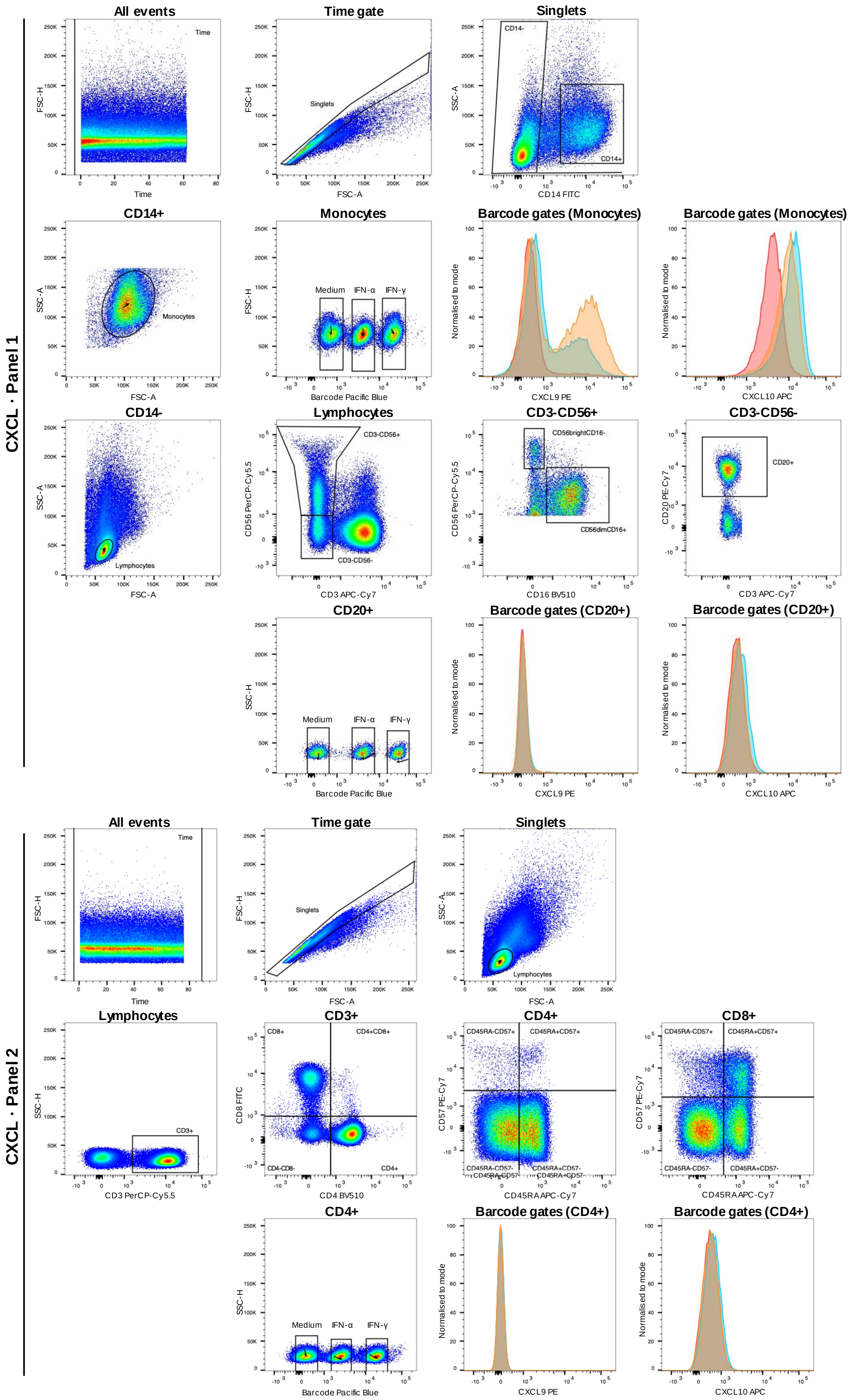

S7C Fig. Gating strategy for CXCL panels. Histograms for CXCL: Medium (red), IFN-α (blue), IFN-γ (orange).

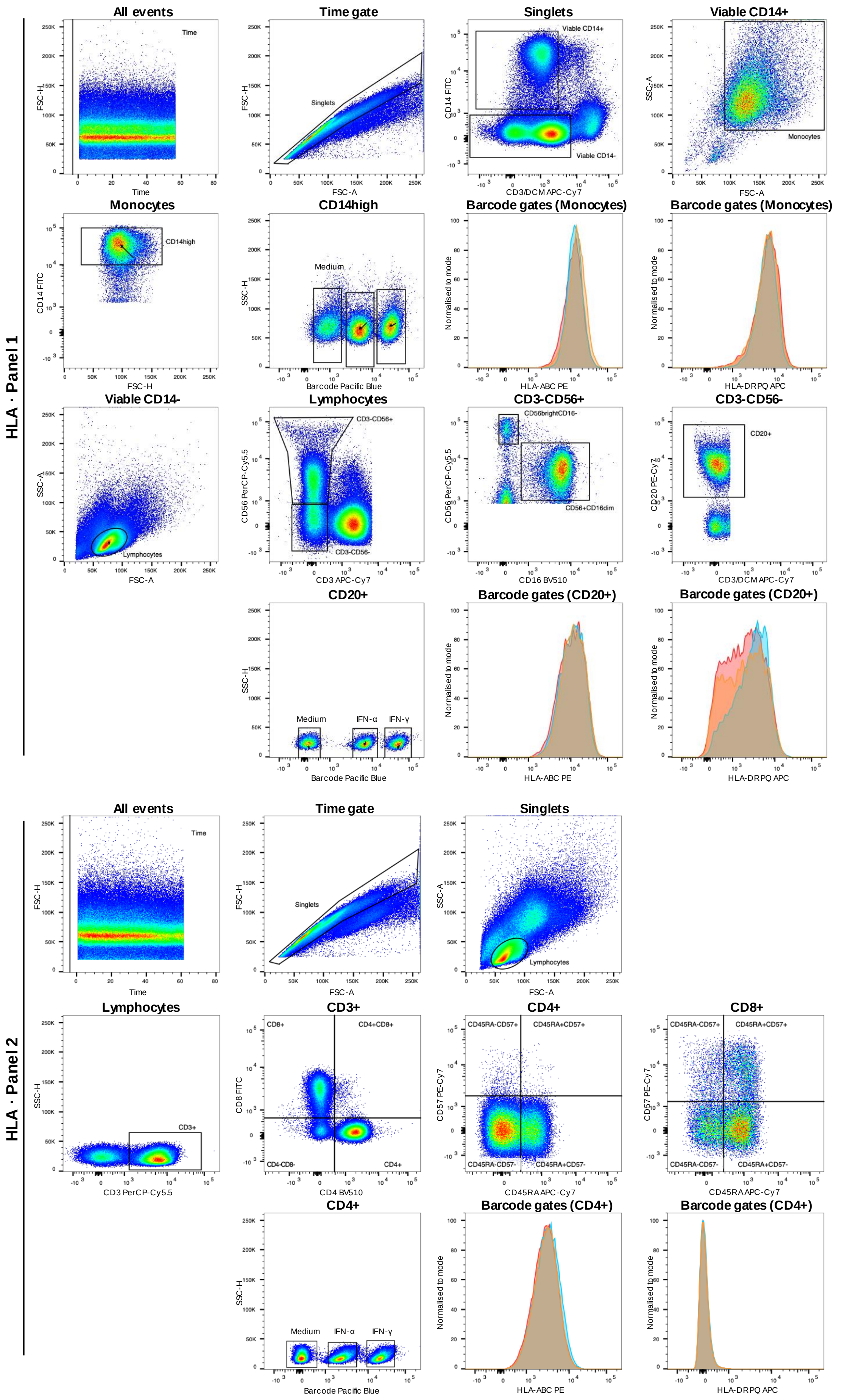

S7D Fig. Gating strategy for HLA panels. Histograms for HLA: Medium (red), IFN-α (blue), IFN-γ (orange). DCM – dead cell marker

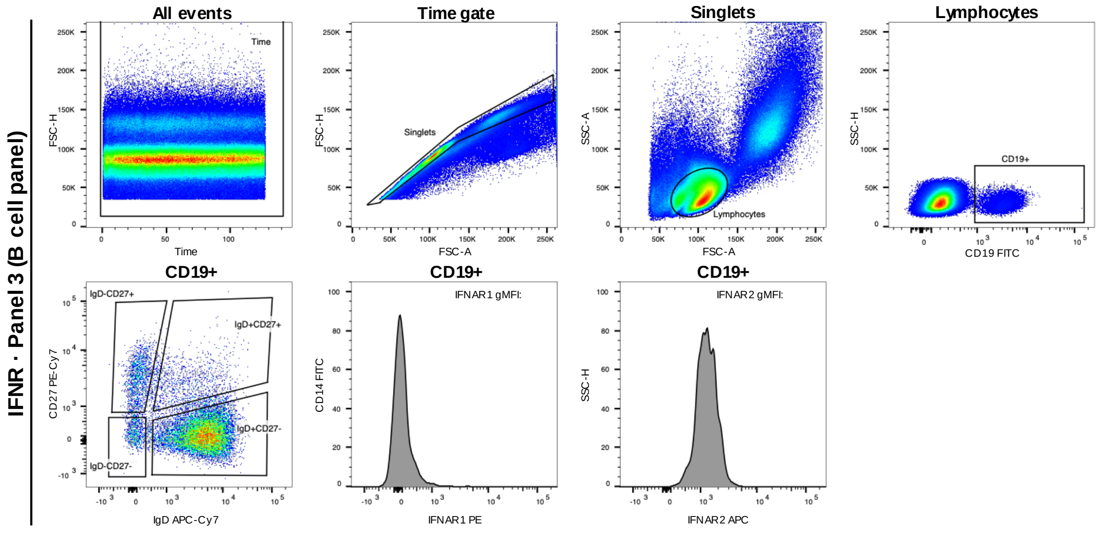

S7E Fig. Gating strategy for B cell subsets
