# Supplementary material for: Function of multiple sclerosis-protective HLA class I alleles revealed by genome-wide protein-quantitative trait loci mapping of interferon signalling
Source: PLoS Genet. 2020 Oct 26;16(10):e1009199. doi: 10.1371/journal.pgen.1009199 (PMC7644105; doi:10.1371/journal.pgen.1009199)
Supplement: S8 Fig — HEK293T cells were transfected with (A) siRNA targeting a sequence shared between IFNAR2b and IFNAR2c ((dTdT-)GCACCATAGTGACACTGAA-dTdT), or (B) plasmids encoding myc-DKK-tagged IFNAR2b or IFNAR2c (#RC201212 and #RC238664, Origene). Cells were stained with anti-IFNAR2 mAb REA124, which recognize both IFNAR2b and IFNAR2c. Stainings for each condition are compared to non-transfected cells. (PDF) [file pgen.1009199.s008.pdf]

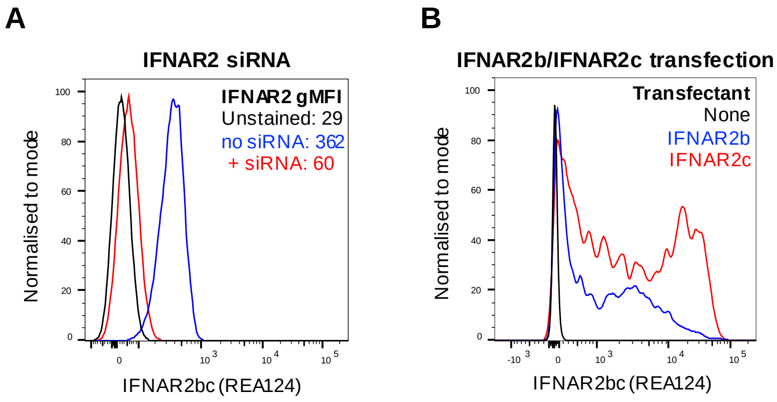

S8 Fig. Validation of the monoclonal IFNAR2 antibody REA124. HEK293T cells were transfected with (A) siRNA targeting a sequence shared between IFNAR2b and IFNAR2c ((dTdT-)GCACCATAGTGACACTGAA-dTdT), or (B) plasmids encoding myc-DKK-tagged IFNAR2b or IFNAR2c (#RC201212 and #RC238664, Origene). Cells were stained with anti-IFNAR2 mAb REA124, which recognize both IFNAR2b and IFNAR2c. Stainings for each condition are compared to non-transfected cells
